# Interactions Among MWCNTs, an Air-Entraining Agent, and Superplasticizers in Lightweight Cementitious Materials

**DOI:** 10.3390/ma19173598

**Published:** 2026-08-24

**Authors:** Ina Pundienė, Jolanta Pranckevičienė

**Affiliations:** Institute of Building Materials, Vilnius Gediminas Technical University, Saulėtekio al. 11, 10223 Vilnius, Lithuania; jolanta.pranckeviciene@vilniustech.lt

**Keywords:** multi-walled carbon nanotubes, superplasticizers, air-entraining agent, zeta potential, rheological properties

## Abstract

This study examined the combined effects on highly foamed cementitious materials of varying concentrations of multi-walled carbon nanotubes (MWCNTs), an air-entraining agent (AEA), and three superplasticizers (SPs): a lignosulfonate-based superplasticizer (SP-LS), a polyacrylate-based superplasticizer (SP-PA), and a polycarboxylate ether-based superplasticizer (SP-PCE). Setting time, semi-adiabatic exothermic-temperature (EXO) profile tests, zeta potential analysis, pH and electrical conductivity (EC) measurements, and foam stability were used to assess the suspensions and pastes. Adding up to 1.5% MWCNTs to an alkaline air-entraining agent (AEA) and SP-LS mostly shifts the zeta potential toward a negative value and stabilizes the suspension and foam. When MWCNTs and SP-LS were added to the foamed paste, the initial viscosity dropped by 12.1% to 7%, whereas with SP-PA and SP-PCE it dropped by 20–48% and 18–50%, respectively. Adding MWCNTs and SP-LS to the foamed paste mostly decreases the density. Using SP-LS is very helpful for interactions between AEA and MWCNTs, thereby stabilizing the air bubble walls. This study underscores the importance of pH and EC of the AEA and SP in affecting the cement paste’s hydration process.

## 1. Introduction

Developing energy-efficient construction materials, such as lightweight concrete for non-structural building elements, offers significant energy-saving and ecological benefits [1,2]. Foamed lightweight concrete is also becoming more popular because it offers better soundproofing [3,4], improved fire resistance due to air bubbles within its microstructure compared to conventional concrete [5], and better thermal insulation [3,6,7,8]. Air-entrained admixture (AEA) is usually used as a foaming agent in such concrete. In concrete, there are four essential conditions for air stability and development: (1) the existence of air; (2) a decrease in pressure on the surface at the border between the air and water; (3) enough strength and flexibility of the layer at the air/water contact to withstand significant thinning during disruption; and (4) enough viscosity of the medium to offset bubble instability from floating and forming [9]. The air gap structure of foam concrete is an essential characteristic that affects its properties, such as strength, density, and durability. Increased resilience to freeze–thaw deterioration and improved cohesiveness (reduced bleed and segregation) of concrete mixtures are two advantages of entraining air in conventional concrete [10,11].

In cement paste, AEA forms a system of microscopic, nearly spherical, air bubbles during mixing. Because the bubbles are mainly larger than 0.01 mm in diameter, with a significant percentage falling below 0.003 mm, they are larger than the capillary pores [12]. The porosity of such concrete can vary significantly based on the nature of AEA utilized, the intensity and duration of mixing, and other factors, because air bubbles in cement paste are naturally brittle. By concentrating on the air–water interface, AEA decreases water’s surface tension because the hydrophilic center of an AEA monomer appears to be in the water. In contrast, the hydrophobic tail, usually represented as a hydrocarbon chain, traps air bubbles that try to escape from the water [13,14]. Charged AEA molecules cover the bubbles, pushing them apart. This prevents bubbles from combining and ensures that the entrained air is evenly and steadily distributed [11,15]. In cement-based materials, ionic AEAs, which are adsorbed on the oppositely charged zones of binder particles, actually generate an inorganic shell around the voids, forming a bridged structure with the air bubbles and thereby increasing the material’s cohesiveness [14] due to the way that cement, as well as additional solid particles in the mix, connect to AEA [13]. Because concrete contains many components, including chemical mixtures, these components may interact with one another, thereby limiting or enhancing their effectiveness. It is hard to imagine making cementitious materials today without viscosity modifiers or plasticizers (SPs) that can reduce water content by 5% to 30% [16]. Some studies show that combining different types of SPs enables the control of the foaming process in cement paste with AEA [17,18].

Meanwhile, it is unknown how AEA affects the hydration process of cement paste. According to some studies, the addition of SP reduces AEA efficiency by causing larger air bubbles to accumulate in the framework [19,20]. Studies on the impact of SP quantity at an identical AEA level showed that although more SP makes the paste more workable [21,22], it also tends to decrease the air amount. Due to the negative charges generated by SP’s adsorption on cement particles, which prevent AEA from adhering to them, the air amount usually decreases when SP is present [23,24].

Due to chemical incompatibility, it is difficult to determine whether AEA is suitable for the SP concrete mix. It is unclear how AEA and SP interact; however, mixtures containing SP typically need greater AEA dosages to achieve the required air volume [25,26,27]. Air quantity is also affected by blending duration and intensity, as well as by the inclusion of AEA and SP sequences [22,28].

According to our earlier research, AEA has the least impact on the early setting time of cement paste compared to other forms of SP. In contrast, SP increases the initial setting time by roughly 25% and combining AEA with SPs further extends it. Furthermore, studies have shown that AEA dramatically lowers cement paste’s fluidity. The research [9] concluded that the presence of AEA decreases the slump of cement paste.

Various nanoscale additives have been investigated for modifying cementitious materials, including nano-silica [29], graphene oxide [30], cellulose nanofibers [31], carbon nanofibers [32], and mineral nanowhiskers [33,34]. They interact with cement minerals in very different ways. While cellulose-based nanofibers largely affect rheology and water retention, nano-silica principally contributes through particle-packing and pozzolanic effects. Because of their high aspect ratios and tendency to form interconnected filamentous structures, carbon nanofibers and multi-walled carbon nanotubes (MWCNTs) may affect viscosity, particle interactions, and possibly the stability of air-paste interfaces. But there are drawbacks to MWCNTs as well, such as aggregation, difficulties dispersing, and comparatively high cost. Therefore, rather than assuming superiority over other nano-additives, their value in foamed cementitious systems should be assessed in terms of fresh-state stabilization.

Nowadays, the use of MWCNT additives enhances the physical and mechanical properties of cementitious materials [35,36]. However, the trend of using MWCNTs to impart distinctive features to concrete, especially lightweight concrete, is still in its initial stages. Among these studies, investigations of nano-additives and chemical admixtures are limited. Most studies focus on the effect of MWCNTs on cementitious materials when SPs are present.

Researchers have also conducted studies on how different types of SP, such as lignosulphonates (LS), polycarboxylates (PCE), and polyacrylates (PA), interact with MWCNTs [37]. For cement pastes containing a selected MWCNT additive content (0.005%), SP-LS reduced the electrical conductivity (EC) by almost 30%, while SP–PCE and SP-PA reduced the EC by 18%. The fluidity of cement paste containing SP-PCE and SP-PA drops by 20% and 25%, respectively, due to SP’s effect on the cement paste’s EC values. On the other hand, SP-LS raises the paste’s viscosity by about 51%. It has been determined that the formation of ettringite increases paste viscosity with SP-LS, thereby reducing the duration of the EXO maximum by 20%. From what we observe, without conducting an in-depth study, it becomes more challenging to forecast the rheological behavior and hydration progress of cement paste as more admixtures and additives are used. However, research on the effects of MWCNTs and AEA on the cement hydration process is minimal. A few studies demonstrate that different types of AEA, whether protein-based or synthetic, exhibit varying effects (behaviors) on cement paste when combined with a superplasticizer [38,39]. Researchers have found that when AEA and SP are combined, AEA enhances SP’s effect and significantly alters the air–water interface. This results in greater workability and higher air content than concrete containing only one of the two admixtures [40].

Much research has been conducted on how various SPs behave in cement paste [41]. Still, the precise relationships among MWCNTs, AEA, and different SP types in cement paste remain unknown and warrant further investigation. Only a handful of references consider the surfactant chemical structure and foaming behavior in cement-like environments.

Researching how MWCNTs interact with AEA and how various types of SP impact the foam suspension stability, foam index, and rheological characteristics of foamed cement pastes, as well as their impact on the exothermic reaction temperature and time during cement hydration, is therefore crucial to the development of MWCNT use in light concrete with foam. Therefore, before deciding on the optimal quantity of MWCNTs in the foamed paste with SP, well-reasoned recommendations and further research are crucial.

In our previous studies, we investigated [12,37] the specific impacts of chemical admixtures or MWCNTs on foam stability and cement-paste rheology. Nevertheless, the combination of MWCNTs and AEA response in the presence of three chemically distinct superplasticizers was not systematically compared in earlier studies across suspension stability, foam retention, fresh-paste density, viscosity evolution, setting time, and semi-adiabatic temperature profiles. The novelty of the present study is therefore the integrated comparison of SP-LS, SP-PA, and SP-PCE within the same MWCNTs–AEA system and under consistent mixture and testing circumstances, and new results confirming that the SP-LS system behaves differently than SP-PCE and SP-PA, and the relationships between pH, EC, dispersion and foam stability being presented as a complex interaction, rather than a single isolated effect.

The study’s findings from various techniques are beneficial for determining the dosage of MWCNTs, SP type, and AEA to use in lightweight concrete, and its potential applications can be extended to non-structural lightweight elements, void-filling materials, insulating cementitious layers, and other specialized composites in which fresh-state foam stability is important.

The main goal of adding MWCNTs to the foamed cementitious system was to see if their high-aspect-ratio filamentous structure affected suspension stability, foam retention, fresh-paste density, viscosity, setting, and early-age temperature evolution in the presence of AEA and various SPs.

## 2. Materials and Methods

### 2.1. Materials

#### 2.1.1. Ordinary Portland Cement

Ordinary Portland Cement (OPC) CEM I 42.5 R, produced in Lithuania by the local manufacturer “Akmenes cementas (Naujoji Akmenė, Lithuania),” complies with EN 197-1 standard [42] requirements. The cement’s mineral composition is as follows: 56.64% C_3_S, 16.72% C_2_S, 8.96% C_3_A, and 10.59% C_4_AF. It has a specific surface area of 4200 cm^2^/g and a bulk density of 1.1 g/cm^3^. The size of OPC particles reaches 100 μm, with an average particle size of 15–30 μm. The size distribution of particles was performed using a “CILAS 1090” (CILAS, Orléans, France). This cement requires 2.33 h for the initial setting and 3.17 h for the final setting. Its alkali content goes up to 0.8%.

#### 2.1.2. Multi-Walled Carbon Nanotubes

The pellets GRAPHISTRENGTH CW2-45 contain MWCNTs with a purity greater than 90% and a concentration of 45% by weight of the mixture. Carboxymethylcellulose, comprising 55% of the mixture by weight, disperses these MWCNTs. ARKEMA, a French company (Colombes, France), provides the pellets. The manufacturer did not provide a complete nanotube length and diameter distribution; this is now acknowledged as a limitation.

#### 2.1.3. Air-Entraining Agent

Unger Fabrikker AS, located in Fredrikstad, Norway, manufactures UFAPORE TCO (Alpha Olefin Sulphonate/Sodium Alkenes Sulfonate) air-entraining agent (AEA). AEA is a white powder based on sodium alkenes sulfonate. According to the manufacturer, pH at 20 °C varies from 7 to 9. Bulk density is 1.30 ± 0.05 g/cm^3^. Max. alkali content is ≤1.0%. Dry solids content is 92 ± 2% and Max. sulphate content is ≤5.0%.

This additive facilitates the formation of small pores within the cement matrix. According to studies [43,44], 0.03% AEA is the optimal concentration to maintain strength properties during testing. That is within the manufacturer’s recommended range of 0.01–0.06% of the binder’s mass for AEA content in the cement matrix [18].

#### 2.1.4. Superplasticizers

The following three different types of SP were used in the research: polymer-based synthetic polycarboxylate ester SP-PCE, polyacrylate-based SP-PA manufactured by BASF (Trostberg, Germany), and modified lignosulphonate SP-LS manufactured by STACHEMA LLC (Kolín, Czech Republic). SPs were used in a liquid state. Molecular weight of SP-PCE is 51 g∙mol^−1^, Max. alkali content—≤0.1%; molecular weight of SP-PA is 39.4 g∙mol^−1^, Max. alkali content—˂2.50%; and molecular weight of SP-LS is 35 g∙mol^−1^, Max. alkali content—≤6.0%.

All SPs were liquid, with active substance contents in a water solution of 27% for SP-PCE and SP-PA and 31% for SP-LS. The measurements were conducted at 20 °C, as shown in Table 1 and Table 2, to evaluate the effect of 0.2% SP and AEA on the EC and pH values of a 100 g distilled water solution.

### 2.2. Design of Water Suspensions and Cement Paste

#### 2.2.1. Design of Water Suspensions

The selection of MWCNTs dosages was guided by practices documented in prior research [45]. The five nominal active-MWCNTs dose levels (0.094, 0.188, 0.375, 0.75, and 1.5% by cement mass) were chosen as a roughly geometric progression, with each level being almost double the level before it. The goal of this design was to restrict the number of trial combinations while covering a wide concentration range. The range was selected to capture both low-dosage dispersion effects and potential high-dosage agglomeration or rheological constraints, and it was based on earlier research [31,38]. The assumption of a monotonic response was not used in selecting the levels. The tested system has competing chemical and physical impacts, as indicated by the non-monotonic responses. As a result, rather than being an optimization research, the current dosage series should be viewed as a screening design. All MWCNT percentages reported in Table 2, Table 3, Table 4 and Table 5 refer to the calculated mass of active MWCNTs relative to the mass of cement, rather than to the total mass of the commercial granules. Because GRAPHISTRENGTH CW2-45 contains 45 wt.% MWCNTs and 55 wt.% carboxymethylcellulose (CMC), the required granule mass was calculated by dividing the target active MWCNTs mass by 0.45. Consequently, the CMC introduced with the granules increased proportionally with the nominal MWCNTs’ dosage. To create different percentage MWCNTs water suspension mixtures as described in Table 3, different amounts of pellets were weighed: 2.09, 4.18, 8.33, 16.7, and 33.3 g. As mentioned before, 45% of the pellets consist of pure MWCNTs. Accordingly, 0.94 g, 1.88 g, 3.75 g, 7.5 g, and 15.0 g of MWCNTs were used. The remaining amount of pellets consists of CMC: 1.15 g, 2.3 g, 4.58 g, 9.17 g, and 18.33 g, respectively. Dispersion was achieved by pre-soaking the commercial pellets in distilled water at 95–100 °C for 10 min, followed by ultrasonic treatment at 22 kHz and a nominal power of 480 W using a UZDN-2T ultrasonic disperser for 5–6 min in a 200–300 mL container. The resulting MWCNT suspension was then diluted with 900 g of distilled water (determined based on the cement paste’s water-to-cement ratio of 0.27, totaling 1000 g) and blended in a lab mixer for 2 min under forced mixing. Accordingly, the percentage of MWCNTs in the prepared water suspensions is: 0.094; 0.188; 0.375; 0.75; 1.5%. These experimental MWCNTs-infused water suspensions were then allowed to cool to ambient temperature (20 °C). No separate surfactant was used for MWCNTs dispersion because the commercial pellets already contained 55 wt% CMC as a dispersing component.

To prepare the SPs, AEA, and MWCNTs suspensions, we mixed pre-calculated amounts of SPs and AEA (as indicated in Table 3) with 40 g of already prepared MWCNTs suspension.

The first sets (Z-LS, Z-PCE, Z-PA, Z-A, and Z1–Z5) present different SPs, AEA, and MWCNTs solutions with varying concentrations. The next set (Z-A-LS, Z-A-PCE, Z-A-PA) and (Z1-A–Z5-A) introduces an AEA. The last sets include all components (Z1-A-LS–Z5-A-LS, Z1-A-PCE–Z5-A-PCE, and Z1-A-PA–Z5-A-PA) together.

**Table 3:** Composition (%) of suspensions for zeta potential measurements.

Mixture	MWCNTs *	AEA *	SP-LS *	SP-PA *	SP-PCE *
Z-LS	–	–	0.2	–	–
Z-PCE	–	–	–	–	0.2
Z-PA	–	–	–	0.2	–
Z-A	–	0.03	–	–	–
Z-A-LS	–	0.03	0.2	–	–
Z-A-PCE	–	0.03	–	–	0.2
Z-A-PA	–	0.03	–	0.2	–
Z1	0.094	–	–	–	–
Z2	0.188	–	–	–	–
Z3	0.375	–	–	–	–
Z4	0.75	–	–	–	–
Z5	1.5	–	–	–	–
Z1-A	0.094	0.03	–	–	–
Z2-A	0.188	0.03	–	–	–
Z3-A	0.375	0.03	–	–	–
Z4-A	0.75	0.03	–	–	–
Z5-A	1.5	0.03	–	–	–
Z1-A-LS	0.094	0.03	0.2	–	–
Z2-A-LS	0.188	0.03	0.2	–	–
Z3-A-LS	0.375	0.03	0.2	–	–
Z4-A-LS	0.75	0.03	0.2	–	–
Z5-A-LS	1.5	0.03	0.2	–	–
Z1-A-PCE	0.094	0.03	–	–	0.2
Z2-A-PCE	0.188	0.03	–	–	0.2
Z3-A-PCE	0.375	0.03	–	–	0.2
Z4-A-PCE	0.75	0.03	–	–	0.2
Z5-A-PCE	1.5	0.03	–	–	0.2
Z1-A-PA	0.094	0.03	–	0.2	–
Z2-A-PA	0.188	0.03	–	0.2	–
Z3-A-PA	0.375	0.03	–	0.2	–
Z4-A-PA	0.75	0.03	–	0.2	–
Z5-A-PA	1.5	0.03	–	0.2	–

* Contents are expressed as mass percentages of the total suspension mass.

#### 2.2.2. Preparation of Cement Suspensions for the Foam Index Test

For pure AEA testing, 40 mL of water was poured into a 100 mL cylinder, and 0.012 g of AEA and 8 g of cement were added. The exact process was followed using 0.08 g SP for SPs and AEA testing. Varying concentrations of MWCNTs were used for MWCNTs and AEA testing. 40 mL of a specific MWCNT suspension at a specific concentration was mixed with 0.012 g of AEA. While SP, MWCNTs, and AEA were measured simultaneously, 0.08 g of each SP and 0.012 g of AEA were added to the specific MWCNT concentration suspension (Table 4).

**Table 4:** Composition (%) for 40 mL suspensions for foam index and pH measurements.

Mixture	Cement	MWCNTs *	AEA *	SP-LS *	SP-PA *	SP-PCE *
S	20	0.03	–	–	–	–
S-LS	20	–	0.2	–	–	–
S-PA	20	–	–	0.2	–	–
S-PCE	20	–	–	–	0.2	–
S-A	20	0.03	–	–	–	–
S-A-LS	20	0.03	0.2	–	–	–
S-A-PA	20	0.03	–	0.2	–	–
S-A-PCE	20	0.03	–	–	0.2	–
S-M1-A	20	0.03	–	–	–	0.094
S-M2-A	20	0.03	–	–	–	0.188
S-M3-A	20	0.03	–	–	–	0.375
S-M4-A	20	0.03	–	–	–	0.75
S-M5-A	20	0.03	–	–	–	1.5
S-M1-A-LS	20	0.03	0.2	–	–	0.094
S-M2-A-LS	20	0.03	0.2	–	–	0.188
S-M3-A-LS	20	0.03	0.2	–	–	0.375
S-M4-A-LS	20	0.03	0.2	–	–	0.75
S-M5-A-LS	20	0.03	0.2	–	–	1.5
S-M1-A-PA	20	0.03	–	0.2	–	0.094
S-M2-A-PA	20	0.03	–	0.2	–	0.188
S-M3-A-PA	20	0.03	–	0.2	–	0.375
S-M4-A-PA	20	0.03	–	0.2	–	0.75
S-M5-A-PA	20	0.03	–	0.2	–	1.5
S-M1-A-PCE	20	0.03	–	–	0.2	0.094
S-M2-A-PCE	20	0.03	–	–	0.2	0.188
S-M3-A-PCE	20	0.03	–	–	0.2	0.375
S-M4-A-PCE	20	0.03	–	–	0.2	0.75

* MWCNTs, AEA, and SP contents are expressed as mass percentages relative to the cement mass. The MWCNTs value refers to the calculated active MWCNTs content, not to the total mass of the commercial MWCNTs–CMC pellets.

#### 2.2.3. Preparation of Fresh Cement Pastes

Table 5 illustrates the preparation of fresh cement pastes using pre-prepared water suspensions containing MWCNTs. This research involved studying foamed pastes with SPs, with MWCNTs, and, afterward, with SPs and MWCNTs. All pastes had the same water-to-cement ratio (W/C) of 0.27. While keeping the concentrations of AEA and SP constant at 0.03% and 0.2% by cement weight, the mixes varied the MWCNT dosage from 0.094% to 1.5% [41]. These MWCNT dosages mirror the cement’s percentage composition in the mixtures (0.094%, 0.188%, 0.375%, 0.75%, and 1.5%), as listed in Table 4. The measured AEA, SPs, or all together, were incorporated into 1000 g of suspension containing the selected MWCNTs. A vertical testing mixer with a rotating spindle was used to mix fresh cement pastes. The mixing was conducted at a forced speed of 125 rpm for 4 min.

**Table 5:** Composition of cement paste with different admixtures, %.

Mixture	Cement	MWCNTs *	AEA *	SP-LS *	SP-PA *	SP-PCE *	W/C Ratio
CP-A	100	–	0.03	–	–	–	0.27
CP-A-LS	100	–	0.03	0.2	–	–	0.27
CP-A-PA	100	–	0.03	–	0.2	–	0.27
CP-A-PCE	100	–	0.03	–	–	0.2	0.27
CP-M1-A	100	0.094	0.03	–	–	–	0.27
CP-M2-A	100	0.188	0.03	–	–	–	0.27
CP-M3-A	100	0.375	0.03	–	–	–	0.27
CP-M4-A	100	0.75	0.03	–	–	–	0.27
CP-M5-A	100	1.5	0.03	–	–	–	0.27
CP-M1-A-LS	100	0.094	0.03	0.2	–	–	0.27
CP-M2-A-LS	100	0.188	0.03	0.2	–	–	0.27
CP-M3-A-LS	100	0.375	0.03	0.2	–	–	0.27
CP-M4-A-LS	100	0.75	0.03	0.2	–	–	0.27
CP-M5-A-LS	100	1.5	0.03	0.2	–	–	0.27
CP-M1-A-PA	100	0.094	0.03	–	0.2	–	0.27
CP-M2-A-PA	100	0.188	0.03	–	0.2	–	0.27
CP-M3-A-PA	100	0.375	0.03	–	0.2	–	0.27
CP-M4-A-PA	100	0.75	0.03	–	0.2	–	0.27
CP-M5-A-PA	100	1.5	0.03	–	0.2	–	0.27
CP-M1-A-PCE	100	0.094	0.03	–	–	0.2	0.27
CP-M2-A-PCE	100	0.188	0.03	–	–	0.2	0.27
CP-M3-A-PCE	100	0.375	0.03	–	–	0.2	0.27
CP-M4-A-PCE	100	0.75	0.03	–	–	0.2	0.27
CP-M5-A-PCE	100	1.5	0.03	–	–	0.2	0.27

* MWCNTs, AEA, and SP contents are expressed as mass percentages relative to the cement mass. The MWCNTs value refers to the calculated active MWCNTs dosage, not to the total mass of the commercial MWCNTs–CMC pellets.

### 2.3. Methods of Testing

#### 2.3.1. Zeta Potential Measurement

The zeta potential measurements are cited in [46] as a valuable source of information on the stability of the prepared suspension against coagulation. A double layer forms when counterions, with the opposite charge to the particle surface, wrap around a solid particle in a liquid with a surface charge. The sliding plane is the distance above the surface at which counterions cannot move along with the particle as it passes through the solution. Zeta potential (ζ) is the electrical potential at the sliding plane; it is commonly expressed in millivolts (mV). Particle suspensions with higher definite zeta potential values are more stable than those with lower definite values. That is because a higher absolute value strengthens electrostatic repulsion, preventing particles from sticking together and settling.

The zeta potential of different concentrations of freshly prepared MWCNT suspension was determined to evaluate its stability. The influence of superplasticizers and AEA in MWCNT suspensions was evaluated. The effects of the amount, different SPs, and AEA on the suspension were assessed using a zeta potential analyzer, a Mastersizer 2000, and software version 5.54. The unit was filled with the tested suspensions. A measurement time of 10 s was selected, and the general-purpose analysis model was used. Zeta potential was measured in the range of −150 mV to +150 mV. The procedure was performed 3 times for each sample, and average values were used for data interpretation [47]. Results are reported as mean ± standard deviation.

#### 2.3.2. pH and Electrical Conductivity

Electrical conductivity and pH testing were conducted to describe better the behavior of all components used in foamed cement suspensions. The Mettler-Toledo MPC 227 device (Mettler-Toledo, Columbus, OH, USA) (pH sensor InLab 410, measuring precision 0.01; EC sensor InLab 730, measuring range 0 mS/cm–1000 mS/cm) was used to examine the impact of varying SP concentrations on the EC and pH values of suspensions. Applying an electrical field between two electrodes (+ and −) that starts an electric current allows one to assess electrical conductivity. A temperature of 20 °C was used for all measurements. The procedure was performed 3 times for each sample, and average values were used for data interpretation. Results are reported as mean ± standard deviation.

#### 2.3.3. Test of the Foam Index

Foam formation and stability are governed by several coupled parameters, including surface tension, surfactant adsorption, liquid-film elasticity, drainage, paste viscosity, particle wettability, bubble-size distribution, and mixing energy.

There is currently no recognized standard for determining the foam index in the presence of cement, despite numerous studies [14,40,41,43] recommending this test. The purpose of conducting this test is to forecast whether AEA will work well with MWCNTs, SP, and their combinations, including AEA-SP and AEA-MWCNTs, and finally, AEA-MWCNTs-SP, to assess the compatibility of different concentrations of MWCNTs with foamed cement in the presence of different types of SP. The methodology for this test was adopted from the procedures outlined in [48]. For foamed cement with AEA and SPs, 40 mL of water was poured into a 100 mL cylinder, followed by the addition of 0.012 g of AEA, 8 g of cement, and 0.08 g of the chosen SP (Table 3). The cylinder was closed, and all components were shaken for 30 s. The initial foam height was measured immediately and again after 5 min. For MWCNTs and AEA testing, suspensions of varying concentrations were used. 40 mL of a suspension of the specific MWCNTs dosage was mixed with 0.044 g of AEA and then treated as described above. When MWCNTs, SP, and AEA were tested simultaneously, 0.08 g of each SP and 0.012 g of AEA were added to the specific MWCNTs dosage suspension and treated according to the above procedure. In each case, the suspension was shaken for 30 s, and the initial foam height was measured immediately and again after 5 min. The procedure was performed twice for each sample, and average foam volumes were recorded to assess foam stability and surfactant compatibility with cement. Results are reported as mean ± standard deviation.

The initial foam index, the foam index after five minutes, and the relative foam-volume loss throughout this time were used in this study to assess foaming performance. Another indirect measure of retained air was the density of fresh paste. Surface tension, interfacial rheology, drainage rate, and bubble-size distribution were not measured.

#### 2.3.4. Fresh Cement Paste Density Measurements

Tests have been carried out in accordance with [49] to evaluate the wet, uncompacted, and compacted densities of the fresh concrete mixes. That was done by dividing the weight of the partially compacted concrete sample in the container by its capacity, yielding the uncompacted wet density. The compacted wet density, on the other hand, was determined by multiplying the weight of the fully compacted concrete sample by the container width. The procedure was performed 3 times for each sample, and average values were used for data interpretation. Results are reported as mean ± standard deviation.

#### 2.3.5. Viscosity Measurements

The study involved testing the impact of MWCNTs, AEA, and SP on the viscosity of cement pastes. Furthermore, the impact of AEA and MWCNTs on suspension viscosity was examined. A vibro-viscometer SV-10 (A&D Company, Limited, Tokyo, Japan), which has an error rate of 0.01 mPa·s and an operating range of up to 12,000 mPa·s, was used for these tests. The dynamic viscosity of fresh cement pastes was assessed immediately upon production and at 5 min intervals for 30 min. This duration was selected to align with typical timeframes for the placement of cementitious materials. To conduct the mini-slump test, the paste was poured into a Vicat cone measuring 40 mm in height, 70 mm at the top, and 80 mm at the bottom. By lifting the cone, the paste spread more easily. The viscosity measurements were performed on one independently prepared batch per composition; therefore, these results should be interpreted as comparative trends, and no statistical significance is claimed.

#### 2.3.6. Setting Times

Based on the standard test methods outlined in EN 196 [50], setting times for cement pastes with various admixtures and additives were determined. These tests involved inserting a needle into the paste at 10 min intervals. A steady temperature of 20 °C was chosen for all tests. The procedure was performed 3 times for each sample, and average values were used for data interpretation. Results are reported as mean ± standard deviation.

#### 2.3.7. Hydration Characteristics

The semi-adiabatic test recorded the internal temperature evolution of the fresh cement paste following the ALCOA approach [51]. It did not provide a direct measurement of total heat release or heat-flow rate. The measured temperature peak may be affected not only by cement hydration kinetics but also by specimen density, entrained-air content, heat capacity, thermal conductivity, heat loss, and the CMC introduced with the MWCNT pellets. Using 1.5 kg specimens, the heat produced by the exothermic reactions of the cement paste was measured. These specimens were placed in a 10 × 10 × 10 cm textolite mold, which was insulated, and the experiments were conducted at 20 °C. Each specimen had an embedded A-type thermocouple connected to a data-collection device. This setup enabled the recording of temperature changes over time, providing insights into the hydration process. The semi-adiabatic temperature measurements were performed on one independently prepared batch per composition; therefore, these results should be interpreted as comparative trends, and no statistical significance is claimed.

## 3. Results and Discussion

### 3.1. Properties of Suspensions

#### 3.1.1. Zeta Potential

The zeta potential determines the stability of dispersions and the kind and degree of particle-to-particle interface in a dispersed system. The stability of colloidal dispersions can be linked to the level of the zeta potential, thereby rendering it valuable [52]. A large zeta potential indicates stability, or the resistance of the solution or dispersion to aggregation, for molecules and particles that are small enough. The stability of the dispersion is disturbed when attraction surpasses repulsion because of a low zeta potential. High zeta potential colloids are electrically stabilized, whereas low zeta potential colloids tend to flocculate or coagulate. Throughout this investigation, the signed zeta potential value and its absolute magnitude are separated for clarity. An increase in absolute magnitude indicates stronger electrostatic repulsion and possibly better colloidal stability.

**Figure 1:**
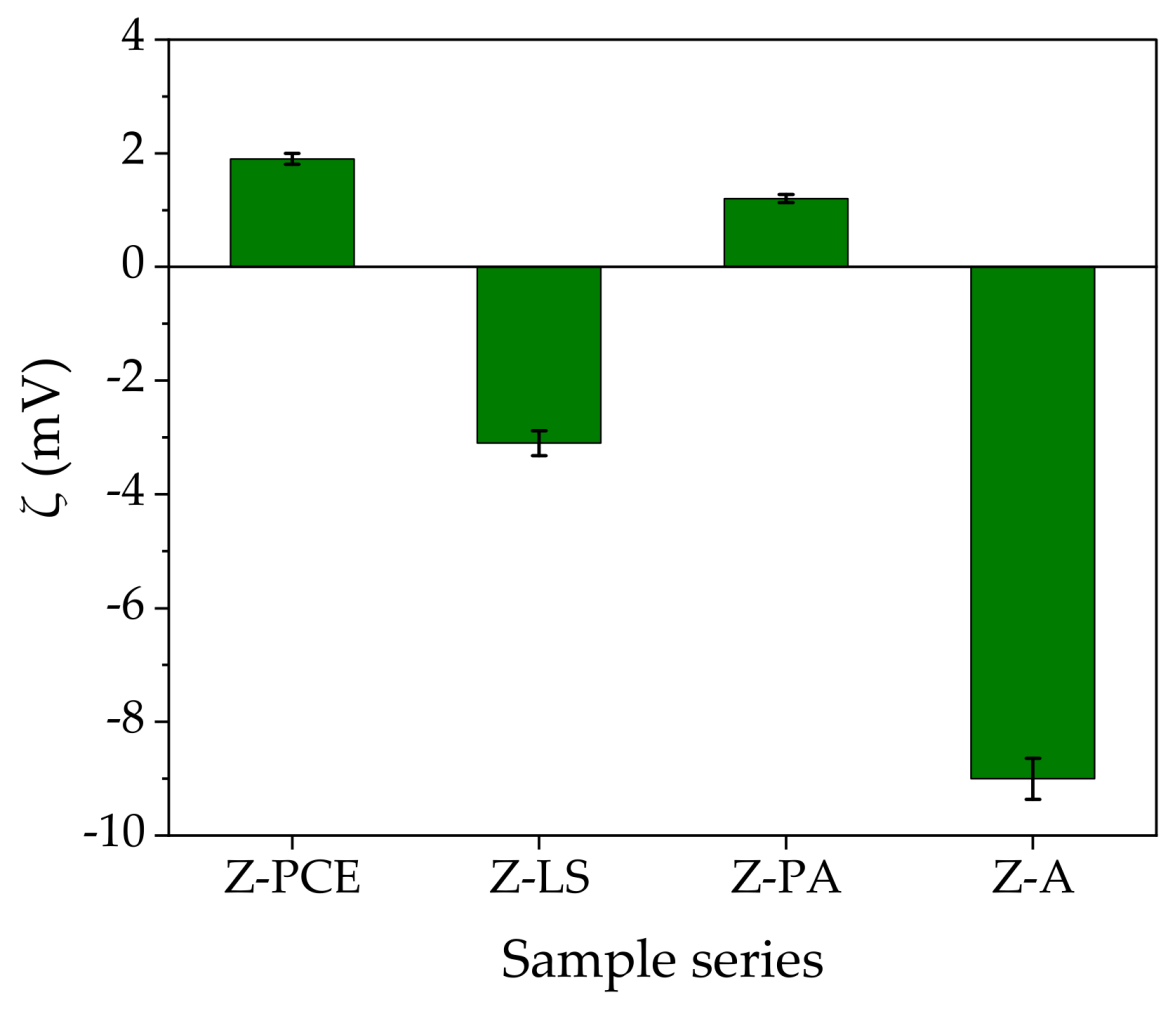
Zeta potential (ζ) values of suspensions with separately used SPs and AEA.

The conditional separation of low-charged and highly charged surfaces can be characterized by a zeta potential value of 30 mV (positive or negative). The colloid’s stability increases with its electrokinetic potential. The stability of colloidal systems is a function of the zeta potential, and therefore, its determination makes it possible to study the mechanism of action of superplasticizers on cement hydration. The zeta potential values of different SPs and AEA were evaluated (Figure 1). Organic admixtures are frequently utilized as dispersants to strengthen the repulsive interactions between cement particles in suspension. These dispersants are usually long-chain polyelectrolytes that carry a charge. These polyelectrolytes adsorb to particle surfaces, alter particle surface charge, increase electrostatic repulsion between particles, and act as a steric barrier to prevent agglomeration [53]. It is interesting to note that SP-PCE and SP-PA, with acidic pH, show small positive zeta potential values.

In contrast, SP-LS and AEA, with more alkaline pH, show negative zeta potential values. Dispersants are usually used as long-chain polyelectrolytes that carry a charge. These polyelectrolytes adsorb to particle surfaces, alter the surface charge of suspended particles, increase electrostatic repulsion between particles, and act as a steric layer to prevent accumulation. Typically, the molecular weights of widely used polyelectrolytes range from 6000 to 15,000 [54]. Higher adsorption capacity can benefit lower molecular-weight polyelectrolytes compared with higher molecular-weight polyelectrolytes [17,55,56]. Because of the carboxylate anion’s surface adsorption (RCOO^−^), carboxylic acids function as low molecular weight dispersants for aqueous suspensions, giving suspension particles a negative surface charge [53]. To cover the particle surfaces with negatively charged species, impart very negative zeta potential values, and keep the non-agglomerating particles in suspension across a broad pH range without agglomeration starting, just a little of a carboxylic acid reagent is all that is needed. The stable state of particle suspensions is probably also maintained by the formation of a steric layer. However, studies have shown [57] that carboxylic acid-based additives impart particles in suspension with negative zeta potential values and electrostatic stabilization. This led to a sharp decrease in zeta potential at specific addition levels, corresponding to the particles’ surface saturation with negatively charged carboxylate groups [57]. On the contrary, as pointed out in [45], introducing SP-LS-based additives leads to a substantial shift in the zeta potential to the negative region.

**Figure 2:**
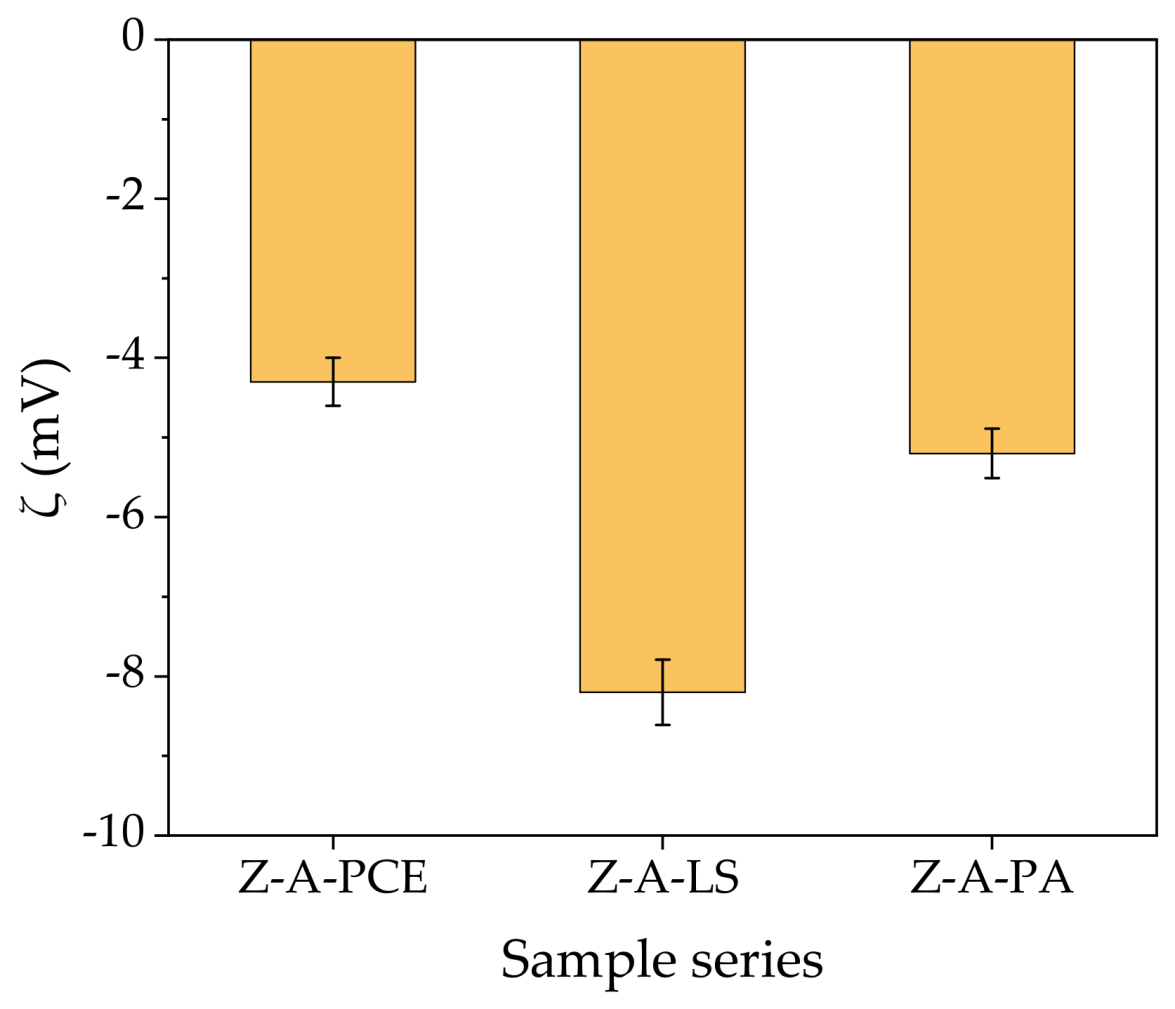
Zeta potential values of foamed suspensions with different SPs.

When SPs were mixed with AEA, the zeta potential values changed (Figure 2). Compared to pure SPs, the presence of AEA increased the zeta potential of suspensions. The highest increase in zeta potential belongs to the mix of SP-LS and AEA. This means that for such a kind of SP, repulsion between particles in the presence of AEA is the highest and increases the system’s stability [45,58].

**Figure 3:**
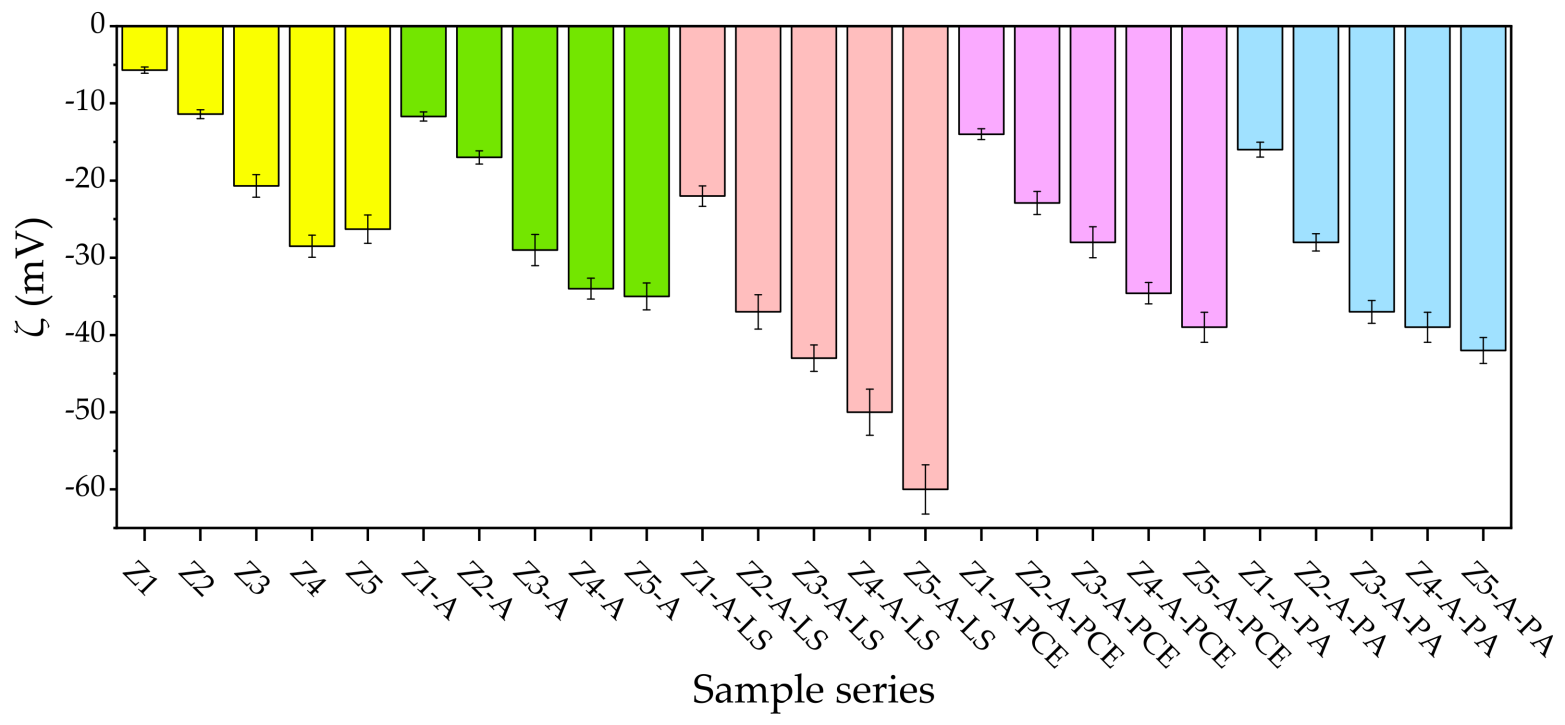
Zeta potential values of MWCNTs suspensions and foamed MWCNTs suspensions with different SPs.

The zeta potential measurements for different MWCNT dosages (Figure 3) showed that increasing MWCNTs from 0.094 g to 0.75% shifts zeta potential values from −5.7 mV to −28.6 mV. At an MWCNTs dosage of 1.5%, the zeta potential value shifts to −26.3 mV. This shift in zeta potential values shows that the highest dosage of MWCNTs can decrease suspension stability. The zeta potential values of AEA solutions with MWCNTs show that the presence of MWCNTs shifts zeta potential values toward more negative values, from −11.7 mV to −35.0 mV. Thus, the signed value became more negative, while its absolute magnitude increased from 11.7 to 35.0 mV, indicating stronger electrostatic stabilization.

This means that increasing the MWCNTs dosage to 0.375% improves suspension stability. The higher MWCNT dosage may be associated with the agglomeration of carbon nanotubes, which did not improve stability [45].

When MWCNTs were used simultaneously with all components (SPs-AEA), the zeta potential in the solution changed. The presence of MWCNTs in the SPs-AEA suspensions increases the zeta potential values but at a different rate, depending on the type of SPs. As reported in the literature [45], SPs with acidic pH show a smaller increase in the absolute zeta potential magnitude than those with alkaline pH. A smaller increase in |ζ| was observed when MWCNTs were used in SP-PCE and SP-PA suspensions—from −14.0 mV to −40.0 mV and −15.7 mV to −42.0 mV. The most visible shift toward negative values in zeta potential is obtained for SP-LS-AEA suspensions. With increased MWCNTs, zeta potential values shift from −22.0 mV to −60.0 mV, corresponding to an increase in |ζ| from 22.0 to 60.0 mV and stronger electrostatic stabilization.

It seems that dispersion is increased in the presence of AEA. The presence of MWCNTs additionally increases repulsion between ions and the system’s stability, especially at higher concentrations, and shifts zeta potential to more negative values. Compared to SPs, AEA visibly shifts the zeta potential toward negative values in suspensions. That means that MWCNTs dispersion with SP-LS, and especially with AEA, may be associated due to the pH of these admixtures being alkaline, which possesses better stability, as reflected in the zeta potential measurement.

In general, a larger absolute zeta potential magnitude usually shows stronger electrostatic repulsion between dispersed particles. In the present study, compositions with a larger absolute zeta potential magnitude often also exhibited improved foam retention, particularly in the MWCNTs–AEA–SP-LS suspensions series. However, this correspondence should not be interpreted as a universal causal relationship. Zeta potential characterizes the dispersed solid phase, whereas SPs and AEA adsorption, surface tension, film elasticity, liquid drainage, bubble coalescence, viscosity, and ionic content and composition additionally govern foam stability.

#### 3.1.2. The Foam Index Test

The inclusion of cement reduced the stability of AEA foam. The presence of calcium ions influences the foaming ability of AEA, and AEA forms an inorganic layer surrounding air spaces [59,60]. The decrease in free AEA molecules can be attributed to adsorption into solid substrates or the association of anionic AEA with the large amount of calcium ions (Ca^2+^) in the solution. Because calcium ions agglomerate anionic surfactants and decrease the quantity accessible to interface adsorption, they raise the surface tension for anionic surfactants [15,61,62,63,64]. Foam stability depends on surface tension and AEA film elasticity [15].

**Figure 4:**
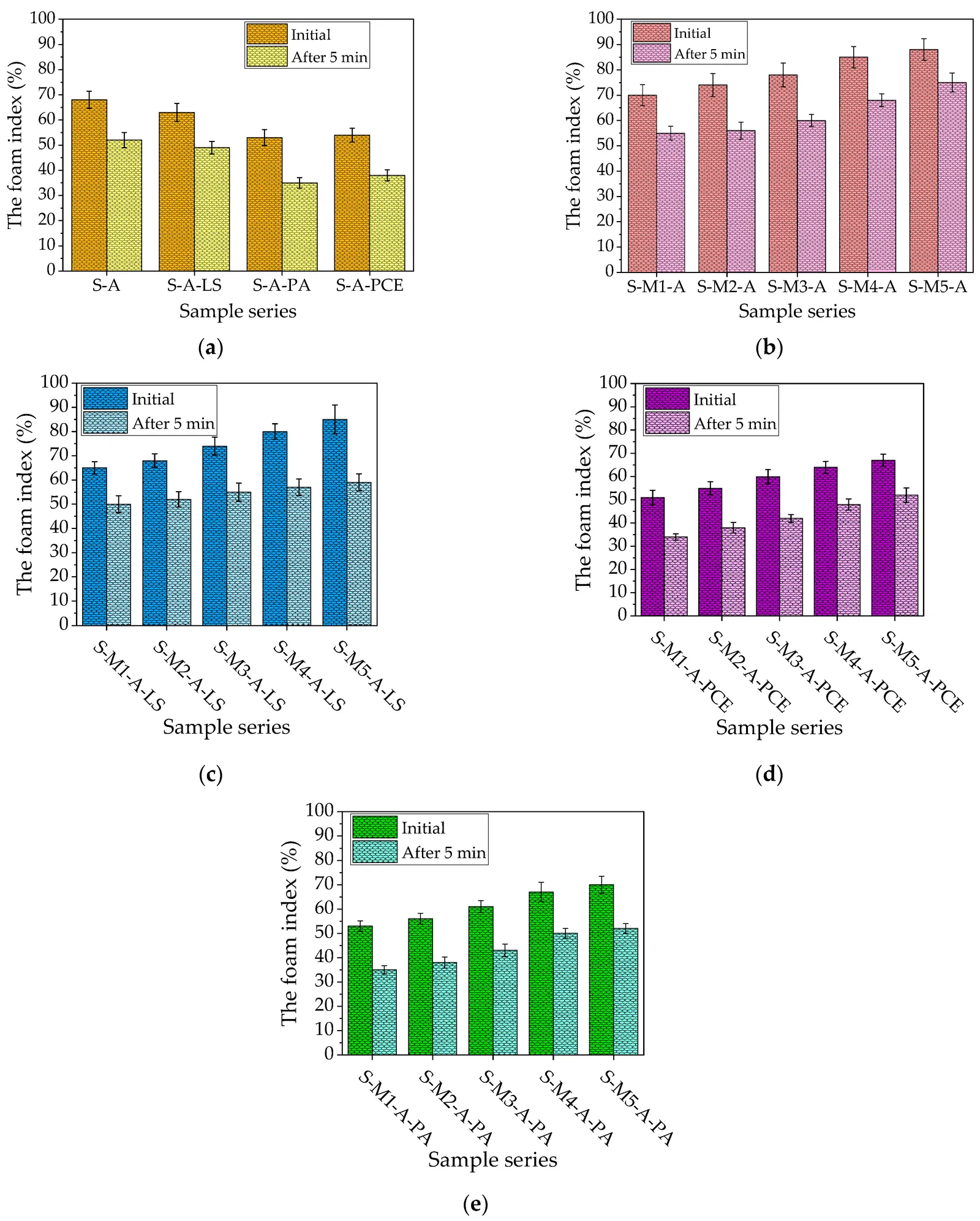
Foam index test results of foamed cement suspension: (**a**) with various SPs; (**b**) with varying MWCNT amounts; (**c**) with SP-LS and varying MWCNT amounts; (**d**) with SP-PCE and varying MWCNT amounts; (**e**) with SP-PA and varying MWCNT amounts.

The research shows that the initial foam volume is the highest in the S-A and S-A-LS compositions. Compared to the S-A composition, SP-PA and SP-PCE reduce the initial foam volume by up to 21% (Figure 4a). After 5 min, foam volume decreases. The S-A-PCE composition exhibits the most notable volume change. The air intake in the composition is known to be hindered by SPs [13]; however, this is primarily observed with SP-PA and SP-PCE superplasticizers. SP-LS superplasticizer exhibits limited volume change due to the sulfate ion, which can interact with cement minerals and promote early hydration. The lignosulphonates accelerate ettringite formation, resulting in rapid paste setting [65]. An increased ettringite generation after around seven minutes of hydration indicates the virtually complete presence of ettringite because lignosulphonates are known to react with C_3_A.

The initial volume of foamed compositions increases significantly with higher dosage of MWCNTs in the suspensions (Figure 4b). In contrast to S-A compositions free of MWCNTs, the starting volume increases by 17–47%. The volume of foam lost after 5 min decreased from 25.1% to 14.9% with increasing MWCNT dosage in the suspension.

**Figure 5:**
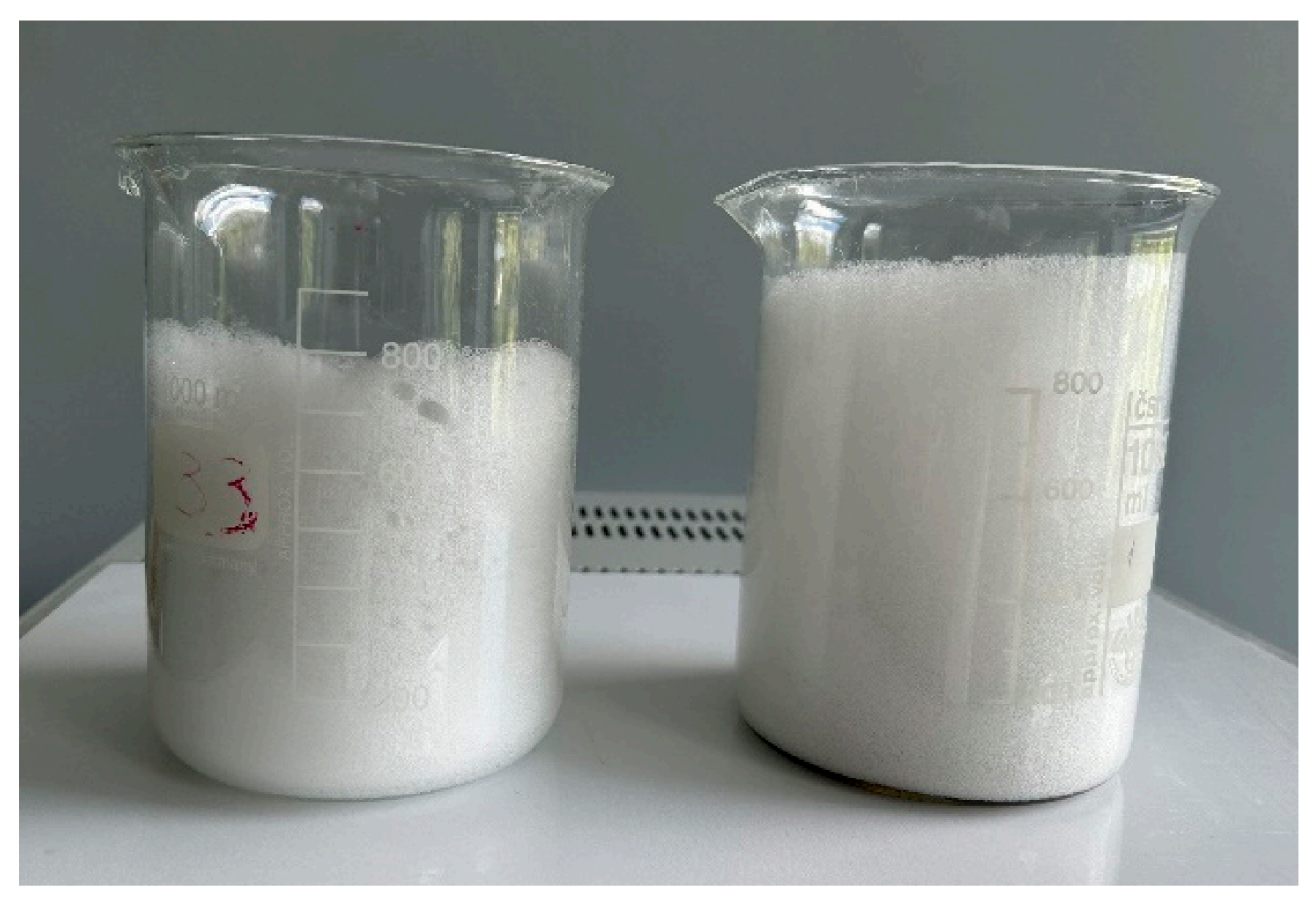
The view of the foamed suspension S-A without MWCNTS (view from the (**left side**)) and foamed suspension S-M5-A with a higher dosage of MWCNTS (view from the (**right side**)).

Compared to the same foamed S-A composition without MWCNTs, the foam volume of the S-M1-A–S-M5-A compositions is 2–31% higher. This result shows the beneficial effect of MWCNTs used with AEA for foam volume and stability. The same amounts of S-A compositions without MWCNTs and S-M5-A were prepared to illustrate these findings. The mixing time was the same. After 5 min, the foam volume was different. As shown in Figure 5, the foam height in the suspension with MWCNTs is higher. It supports the view that MWCNTs facilitate the incorporation of additional air volume into the foam.

The presence of MWCNTs in the foamed composition with SP-LS, compared to the S-A-LS composition, increases the initial volume of foam from 3.2% to 35.6% (Figure 4c). It is evident that the presence of MWCNTs positively influences foam volume because, with the growth of the MWCNT dosage, the starting volume rises. After 5 min, the foam volume loss ranges from 21.5% to 14.1% compared to the initial volume. Compared to the S-A-LS composition, foam volume after 5 min increased from 2.1% to 22.5%. This result shows compatibility among AEA, SP-LS, and MWCNTs, as SPs markedly decrease foam volume and stability. However, another study reports results contrary to those for MWCNTs, SP, and AEA interaction [58]. Researchers find that using a variety of admixtures and MWCNT combinations in concrete samples can decrease the foam index and air content to 42% and 60%, respectively, and that this significant change in the air system can compromise concrete’s freeze–thaw resistance. These results indicate that MWCNTs can minimize the AEA effect. The presence of MWCNTs (Figure 4d) in the foamed composition with SP-PCE increases the initial foam volume from 1.8% to 19.1%. Compared to the S-A-PCE composition, the increased initial foam volume falls within the same range because the small dosage of MWCNTs practically does not affect the initial foam volume. However, compared to the effect of SP-LS in the composition, the increase is much smaller. After 5 min, the foam volume loss is from 28.7% to 22.7% compared to the initial volume. According to research [66], when alkylbenzene sulfonic acid-based AEA was used to disperse MWCNTs, the conclusion was drawn that AEA can disperse MWCNTs, but not as well as polycarboxylate-based surfactant. However, when AEA and sodium deoxycholate surfactants were used together to disperse MWCNTs, it was found that this combination could achieve qualified dispersion and improved hardened cement mechanical properties [67]. Compared to the S-A-PCE composition, the foam volume of MWCNT-containing compositions after 5 min increased from 0.14% to 25.8%. The research indicates that compatibility between AEA, SP-PCE, and MWCNTs is lower than with SP-LS, because the initial foam volume with SP-LS increased up to 2 times compared to that of compositions with SP-PCE. The presence of MWCNTs (Figure 4e) in the foamed composition with SP-PA increased the initial foam volume from 1.75% to 18.6%. Compared to the S-A-PCE composition, the increased starting foam volume is practically the same because a small dosage of MWCNTs does not influence the initial foam volume. Meanwhile, the initial foam volume increases, whereas the effect of SP-LS is much smaller.

After 5 min, the foam volume loss ranges from 1.27% to 26.7% compared to the initial volume and S-A-PCE composition. The research indicates that compatibility between AEA, SP-PCE, and MWCNTs is lower than with SP-LS, because the initial foam volume with SP-LS increased up to 2 times compared to that of compositions with SP-PCE or SP-PA. In conclusion, adding MWCNTs improves foam stability, but the effect depends on the SP employed. SP-LS primarily affects foam stability, whereas SP-PCE contributes less.

#### 3.1.3. The Foamed Cementitious Suspension pH Test

**Figure 6:**
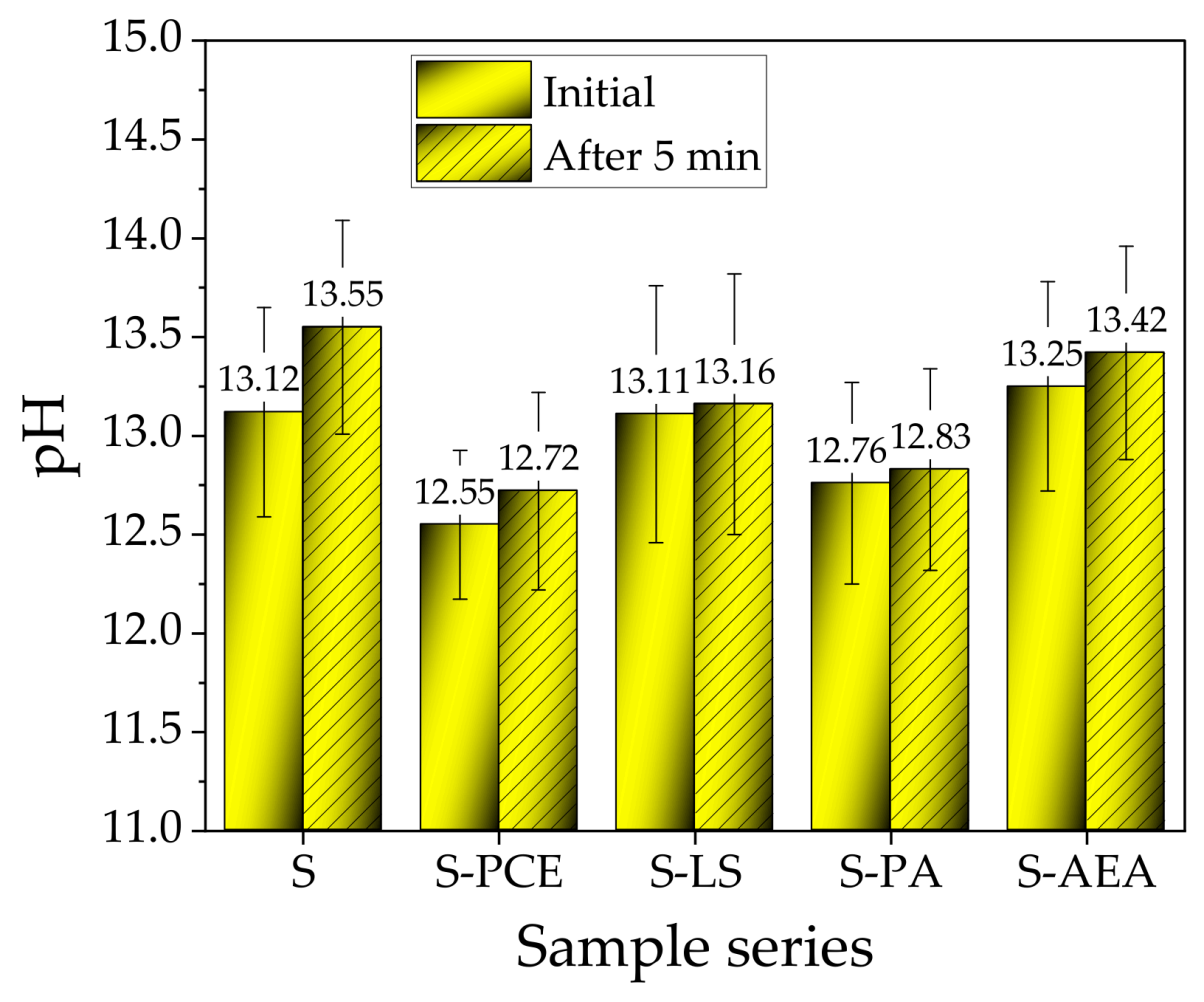
The pH values of cement suspension with different SPs foamed by AEA cement suspension.

The impact of adding MWCNTs to foamed cementitious suspensions containing various SPs was examined (Figure 6). The highest pH value of 13.12 was observed in the cement suspension. The addition of SPs differently influences pH: mainly, pH decreases in SP-PCE and SP-PA, reaching 12.55 and 12.76, because their pH in water solution is acidic, and has less influence on SP-LS and AEA, which have alkaline pH and reach 13.11 and 13.25. After 5 min, as expected, an increase in pH values is observed in all suspensions. pH values increase primarily in pure cement suspension (13.55) than in S-A and S-LS suspensions (13.42 and 13.16), and less in S-PA and S-PCE suspensions (12.83 and 12.72). Comparing with the zeta potential results in Figure 1, it can be observed that there is a certain tendency—SP-LS and AEA, possessing an alkaline pH, show a negative zeta potential region. In contrast, SP-PCE and SP-PA at acidic pH show small values in the positive zeta potential region. As noted in [45], as pH decreases, the zeta potential increases to more positive values.

**Figure 7:**
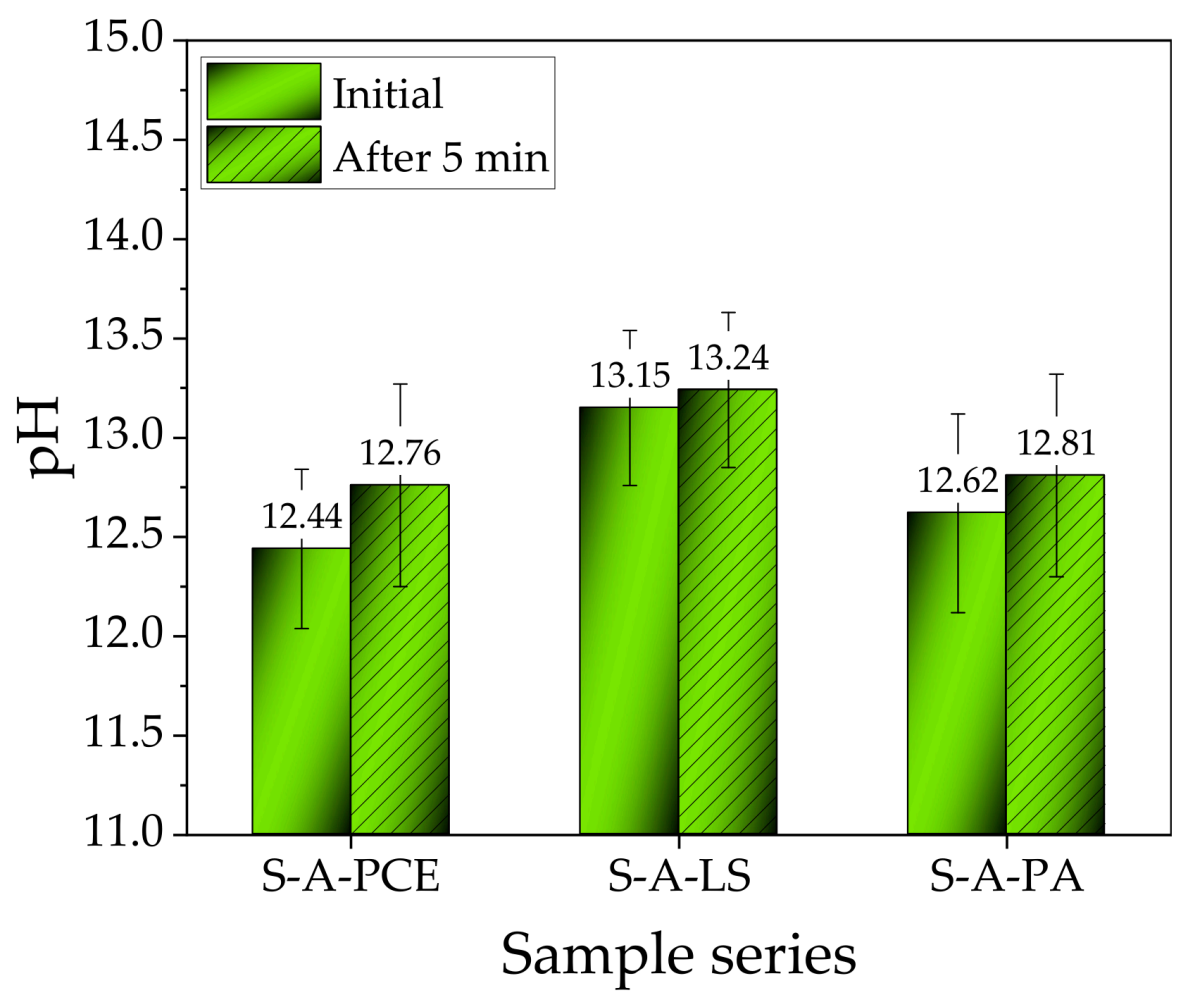
pH values of foamed cement suspension in the presence of different SPs.

When AEA is added to suspensions with SPs (Figure 7), the same tendencies in pH behavior were observed. The presence of AEA slightly increases pH in suspensions. The highest pH values are observed in the S-A-LS suspension. Comparing with the zeta potential results in Figure 2, it can be observed that there is a certain tendency; the presence of AEA in suspensions with SPs changes the zeta potential, and all suspensions’ zeta potential values are found in the negative zeta potential region.

**Figure 8:**
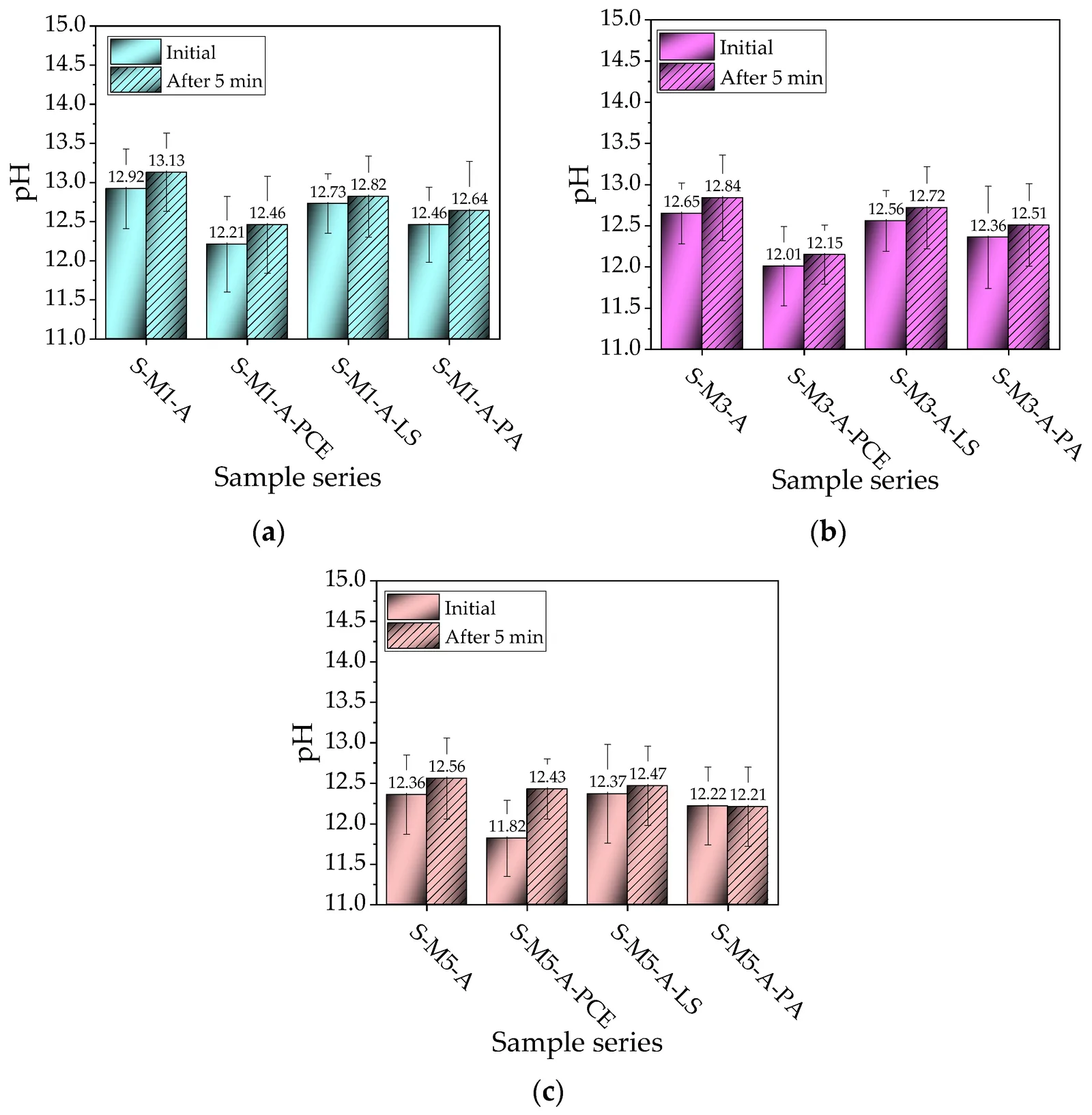
pH values of foamed cement suspension in the presence of different SPs: (**a**) lowest dosage of MWCNTs (0.094%); (**b**) middle dosage of MWCNTs (0.375%); (**c**) highest dosage of MWCNTs (1.5%).

The lowest MWCNT dosage (0.094%) showed a slight decrease in pH, with the MWCNT suspension slightly acidic (pH 6.75; Figure 8a), but the pH values of S-M1-A and S-M1-A-LS remain the highest. S-M1-A-LS suspension zeta potential is the same and the largest. After 5 min, as expected, an increase in pH is observed in all suspensions. pH values increase mainly in S-M1-A and S-M1-A-LS 13.13 and 12.82. In the S-M1-A-PCE and S-M1-A-PA suspensions, pH values are lower—at 12.46 and 12.64. When the middle dosage of MWCNTs (0.375% of MWCNTs) was used, pH values decreased (Figure 8b). The pH of the MWCNT suspension with 0.375 g of MWCNTs was 6.42, which is more acidic, and, as expected, it influenced the suspensions’ pH values more. The pH values decreased from 2.3% to 1.1%, compared to suspensions with the lowest MWCNTs dosage. As in Figure 3, the zeta potential of all suspensions with increased MWCNTs increased. After 5 min, as expected, an increase in pH values is observed in all suspensions. However, the tendencies are the same. S-M3-A and S-M3-A-LS pH values are 12.84 and 12.72; for S-M3-A-PCE and S-M3-A-PA suspensions, pH values are lower than 12.15 and 12.51.

When the highest dosage of MWCNTs (1.5%) was used, the pH values decreased further (Figure 8c). The pH of the MWCNTs suspension with 1.5% MWCNTs was 5.68, further decreasing the suspension pH. The pH values decreased from 2.14% to 3.3% compared with suspensions containing a middle MWCNTs dosage. According to the Figure 3 data, the zeta potential of all suspensions with increased MWCNTs increased markedly, especially for S-M5-A-LS. After 5 min, the pH values increased in all suspensions. S-M5-A and S-M5-A-LS pH values are 12.56 and 12.47; for S-M5-A-PCE and S-M5-A-PA suspensions, pH values are lower than 12.43 and 12.21. It is clear that SP-LS mainly increases the zeta potential in interaction with AEA due to its alkaline nature, and this increase was strengthened during the interaction with MWCNTs, as evidenced by zeta potential measurements and pH data.

In general, pH may affect the ionization of SPs and AEA polymer functional groups, adsorption on cement- or MWCNT-containing surfaces, and the concentration of dissolved cement ions. Consequently, pH can influence both zeta potential and foaming behavior, but it was not independently controlled in the present study.

### 3.2. Properties of Fresh Cement Paste

#### 3.2.1. Paste Volume

**Figure 9:**
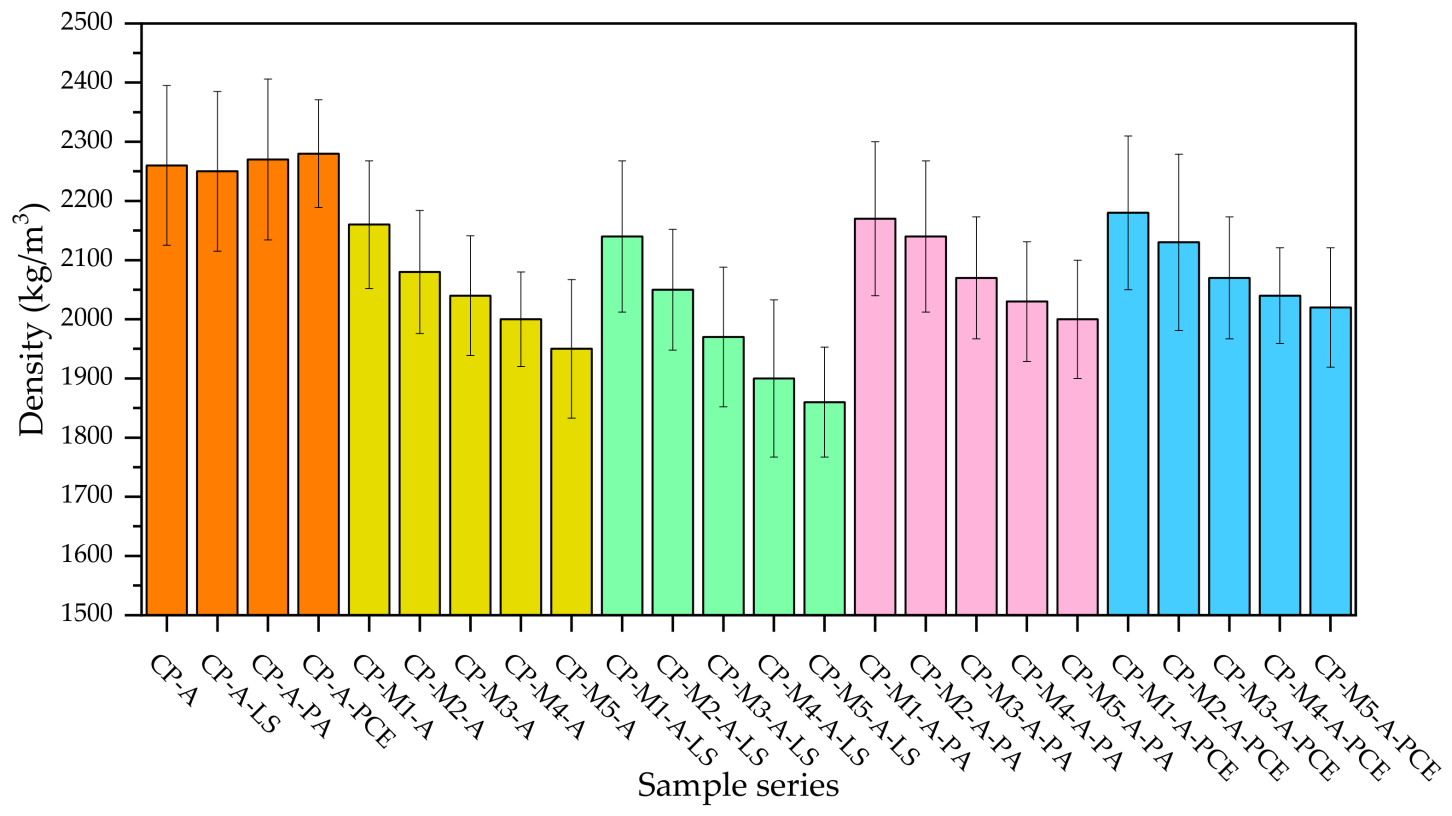
The fresh foamed paste density, depending on different kinds of SPs, different dosages of MWCNTs, simultaneous exposure to SPs, and different dosages of MWCNTs.

Fresh paste density is one of the most critical properties of building materials. The fresh paste density (Figure 9) results showed that the paste with SP-PCE had a higher fresh paste density than that with SP-LS, apparently due to the rapid reaction of sulfate ions with calcium. The increase in MWCNTs in the foamed pure cement paste, as expected, decreased the mass and increased the volume of the paste, reflected in the calculated fresh paste density, which decreased from 2260 kg/m^3^ for the reference to 1940 kg/m^3^ with the highest MWCNT content (about 14.1%). A hypothesized MWCNT-related reinforcement effect on the pores created by the AEA wall surface may be associated with this decrease. It may be similar to a network of nanofibers that supports the stability of the pore walls.

**Figure 10:**
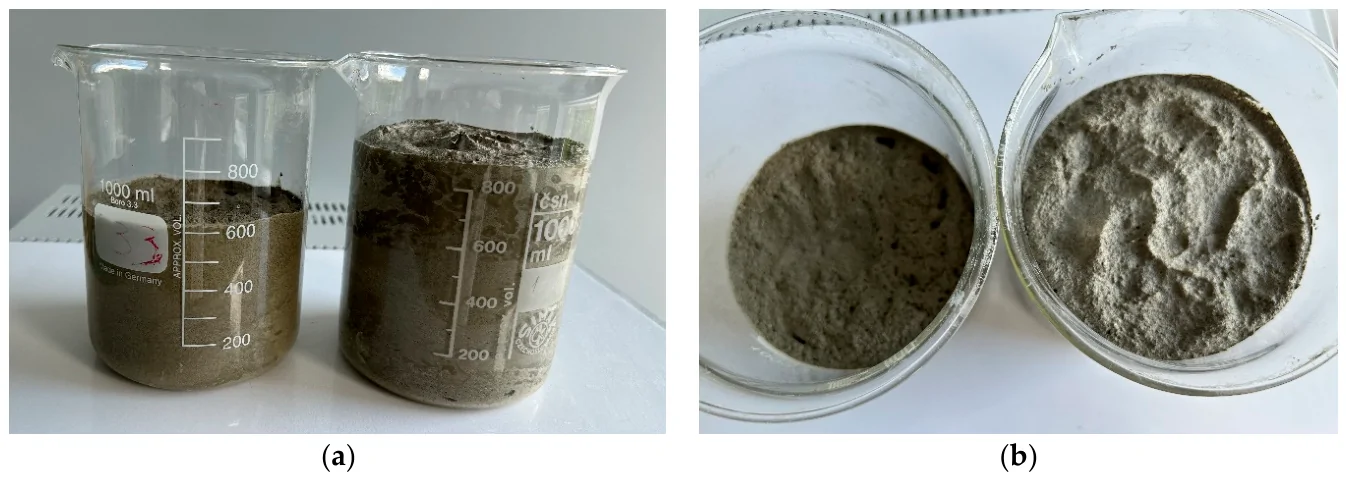
The view of the foamed CP-A fresh paste composition, without MWCNTs (view from (**a**)) and CP-M5-A fresh paste composition with the highest MWCNTs dosages (view from the (**b**)).

**Figure 11:**
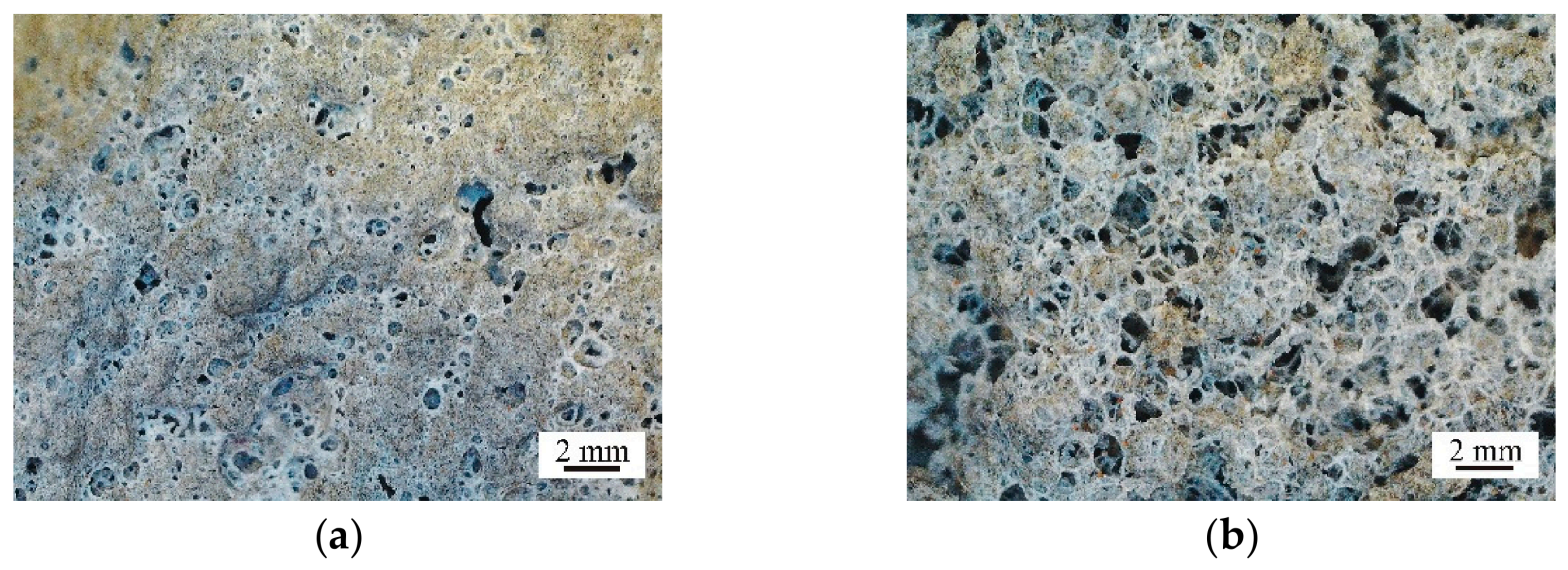
The view of the foamed CP-A composition paste without MWCNTs (view from (**a**)) and CP-M5-A composition paste with the highest MWCNTs dosages (view from (**b**)).

The CP-A composition paste without MWCNTs and the CP-M5-A compositions with the highest MWCNTs content were compared to illustrate these results (Figure 10). However, these surface images (Figure 10 and Figure 11) provide qualitative information only. They do not represent a quantitative pore-size distribution or three-dimensional pore connectivity.

The view shows that the paste volume with MWCNTs is higher than that without MWCNTs. It shows that MWCNTs, when interacting with AEA, can facilitate air entrapment in the cement paste and form a more stable bubble structure. The appearance of pores and bubbles in the pastes is presented in Figure 11. The macrostructure studies of the paste surface showed that the amount, size, and shape of pores in the paste differ in the composite samples depending on the AEA and MWCNTs ratio. The number, size, and morphology of the visible voids differed between the MWCNTs-free and MWCNTs-containing pastes.

The number of voids on the surface of the paste with MWCNTs is about 5 times higher than in the MWCNTs-free paste. The void shapes in the MWCNT-free paste are mainly spherical, whereas in the MWCNTs-containing paste, the voids are connected in a honeycomb shape. The size of the voids can vary from 0.1 to 1 mm. It seems that in the paste with MWCNTs, the voids are larger. This observation is consistent with the results provided in studies [68,69].

When the foamed paste with MWCNTs is added to SP-LS, it may be observed that the volume of paste rises and fresh paste density decreases from 2140 kg/m^3^ to 1860 kg/m^3^, respectively, by 3.1–5.6%, compared to foamed pure cement paste. When SP-PCE and SP-PA were employed, the increase in paste volume was less than that of SP-LS. With an increased dosage of MWCNTs, the density decreased from 2170 kg/m^3^ to 2030 kg/m^3^.

**Figure 12:**
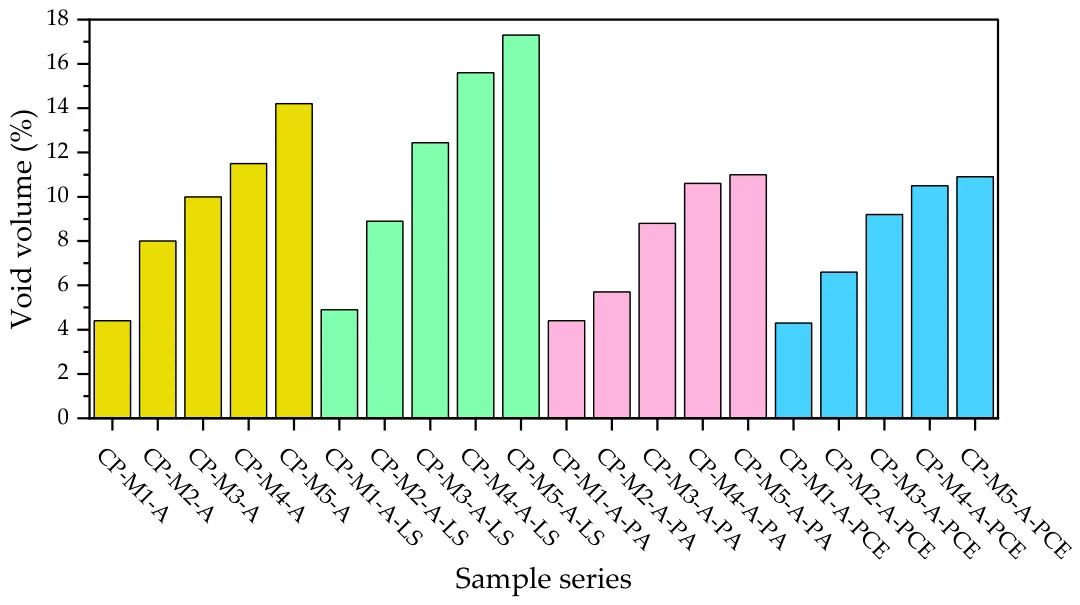
The void volume (%) in the foamed paste, depending on different kinds of SPs, different dosages of MWCNTs, and simultaneous exposure to SPs and different amounts of MWCNTs.

The void volume percentage of foamed paste, calculated by dividing the density of foamed pastes with additives by plain foamed cement CP-A density, is presented in Figure 12. When compared to plain foamed cement CP-A (2260 kg/m^3^), the void volume percentage in the foamed paste with maximal addition of MWCNTs reached 14.1, for the same paste with SP-LS 15.7%, and for the same pastes with SP-PCE and SP-PA 10.9 and 11%. We can see that SP-LS, during interaction with MWCNTs, increases paste volume the most. In contrast, other SPs, associated with their lower stability and zeta potential, do not substantially increase paste volume [70]. We can see that LS, in combination with MWCNTs, increases volume the most, whereas other plasticizers do not, apparently due to their lower stability and zeta potential. According to [71], MWCNTs surface surfactants interact with water and cement particles, increasing the yield stress of the cement paste. The conducted density, void volume, and imaging studies confirm the hypothesis presented. The density reduction and the surface photographs are consistent with the hypothesis that MWCNT-containing filaments may contribute to the stabilization of air bubble walls. However, the present observations do not directly demonstrate nanoscale reinforcement or quantify the three-dimensional pore structure.

#### 3.2.2. Dynamic Viscosity of Cement Paste

The dispersion of air bubbles within the cement paste depends on the paste’s viscosity. According to Stokes’ law [72], if the paste viscosity is too low, the air bubbles will rise and escape due to buoyancy. Although some surfactants may stabilize bubbles without particle addition, their primary role appears to be increasing the surface activity of cement particles by adsorbing them and enhancing their hydrophobicity. That has been supported by numerous studies showing that surfactant adsorption onto cement increases particle hydrophobicity [73].

The gradual structural development of the fresh paste is reflected in the increase in apparent dynamic viscosity over time. Following mixing, early hydration products develop, ions are discharged into the pore solution, and cement particles start to reflocculate. Ettringite formation may be a factor in the sharp rise in flow resistance, especially in systems with lignosulfonate-based admixture. In addition to limiting particle and bubble mobility, the MWCNTs–CMC phase may simultaneously create a physically entangled network. The combined effects of hydration, thixotropic rebuilding, polymer adsorption, interactions between nanotube-containing filaments, and the entrained-air structure are thus represented by the measured viscosity evolution.

**Figure 13:**
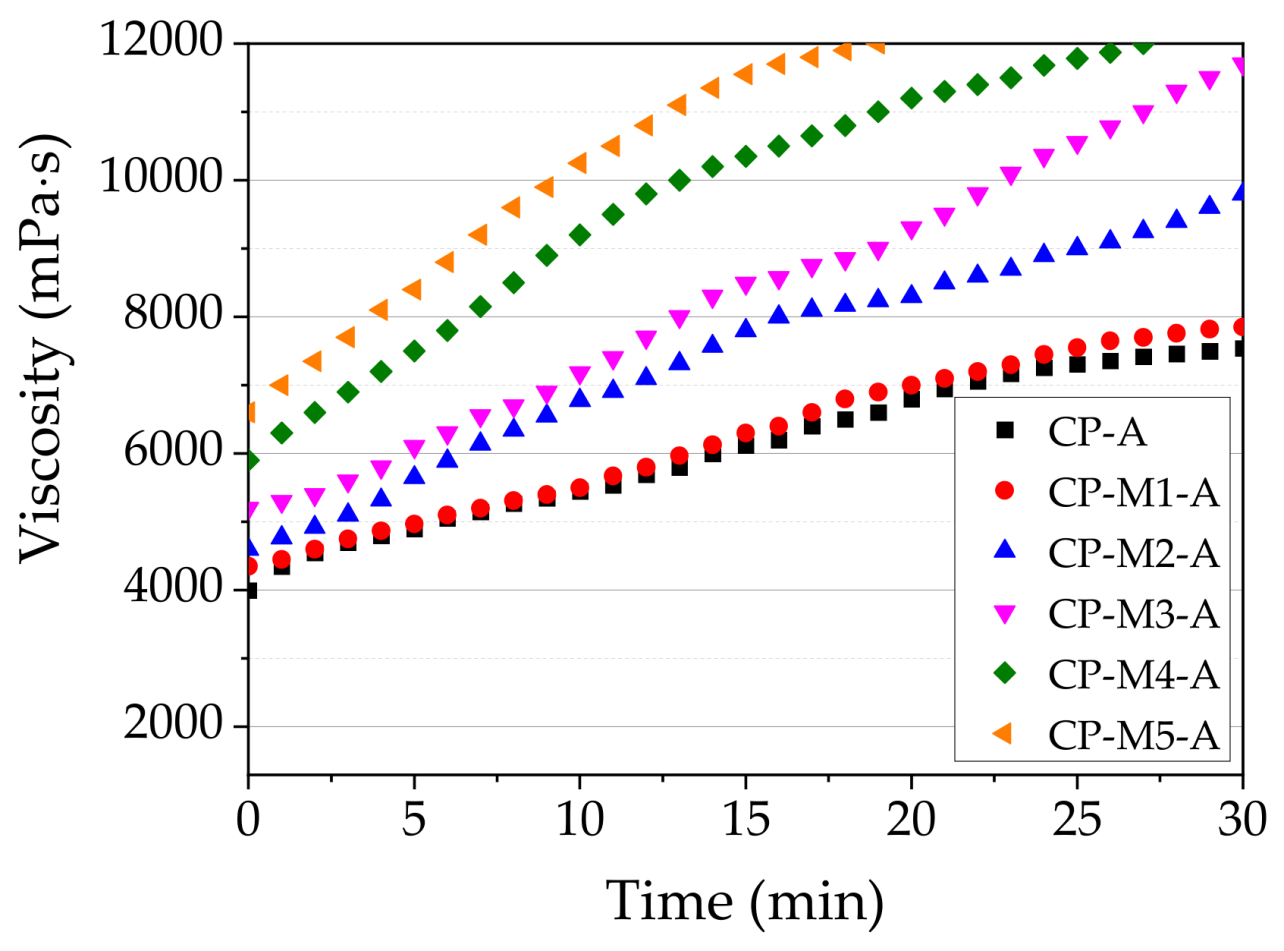
The dynamic viscosity of foamed cement paste with various quantities of MWCNTs.

Various MWCNTs were used to develop a foamed cementitious paste (Figure 13). The initial dynamic viscosity of the foamed paste CP-A is 4000 mPa·s. As the MWCNTs dosage in the paste increases, the initial viscosity also rises significantly—from 4350 mPa·s in the CP-M1-A paste to 6600 mPa·s in the CP-M5-A paste. This means that the MWCNTs viscosity increased by 39.4%. Researchers [14] have noted that, at a given water/binder ratio, entrained air bubbles act as compressible ball bearings, allowing aggregate particles to move relative to one another. However, introducing many air bubbles can decrease plastic viscosity and yield stress when the AEA dosage is high (0.1% of the total mortar’s dry weight).

In our studies, the presence of MWCNTs altered the paste’s viscosity by connecting air bubbles to the network, thereby increasing viscosity. At the end of the testing, the viscosities of the reference CP-A paste and the paste with the lowest MWCNTs dosage were 7540 mPa·s and 7850 mPa·s, respectively. For pastes with higher MWCNTs dosage, the viscosity was significantly higher. Foamed pastes with 0.188% and 0.375% of MWCNTs reached 9800 mPa·s and 11,700 mPa·s, respectively.

The pastes with the highest MWCNTs dosage, 0.75% and 1.5%, reached maximum viscosities of 12,000 mPa·s (the limit of the measuring device) in 26 min and 18 min, respectively.

AEA increases the viscosity of the cement paste. This increase can be attributed to the interaction of anionic AEA with (Ca^2+^) in the cement paste, as detailed in [59]. This interaction reduces the number of free AEA molecules available in the paste. Another contributing factor, as noted in [15,63,64,74], is that calcium ions (Ca^2+^) increase the surface tension for AEA. This is because Ca^2+^ ions cause coagulation of anionic surfactants, reducing the number of surfactants available for interface adsorption.

In conclusion, using MWCNTs in foamed pastes increases viscosity, with a more pronounced effect at higher MWCNT amounts. This phenomenon can be attributed to fewer MWCNT traps and fewer air bubbles when interacting with AEA, which do not prevent a reduction in viscosity. In contrast, pastes with more MWCNTs experience a smaller viscosity decrease associated with the larger air bubbles trapped between MWCNT fibers. MWCNTs may exhibit a significant impact on particle size in foamed paste [75]. MWCNTs filaments interact with every paste component and behave as solid particles in this particular case. The yield stress that governs the foam flow properties increases when cement is incorporated into the paste. Based on reports, foams containing tiny solid particles exhibit granular packings between foam bubbles and increase yield stress. Studies [45,76] indicate that MWCNT dosages higher than 0.05% tend to increase the viscosity of cement pastes associated with MWCNT agglomeration. It is pointed out in the research [9] that to ensure that air bubbles will be incorporated appropriately and maintained in cement paste, several conditions must be met: (1) the surface tension between the air and water must be reduced; (2) the barrier between the air and water must be strong and elastic enough to withstand disturbances and at last the overall paste must be thick enough to prevent air bubbles from rising and escaping. It can be concluded that in pastes with higher MWCNTs dosage (above 0.375%), viscosity is high, making them difficult to use without a viscosity modifier. Therefore, the next step was to investigate the impact of various types of SP on the pastes containing MWCNTs and AEA, including viscosity, and to evaluate the influence of each type of SP on the development of the pastes’ viscosity over time.

**Figure 14:**
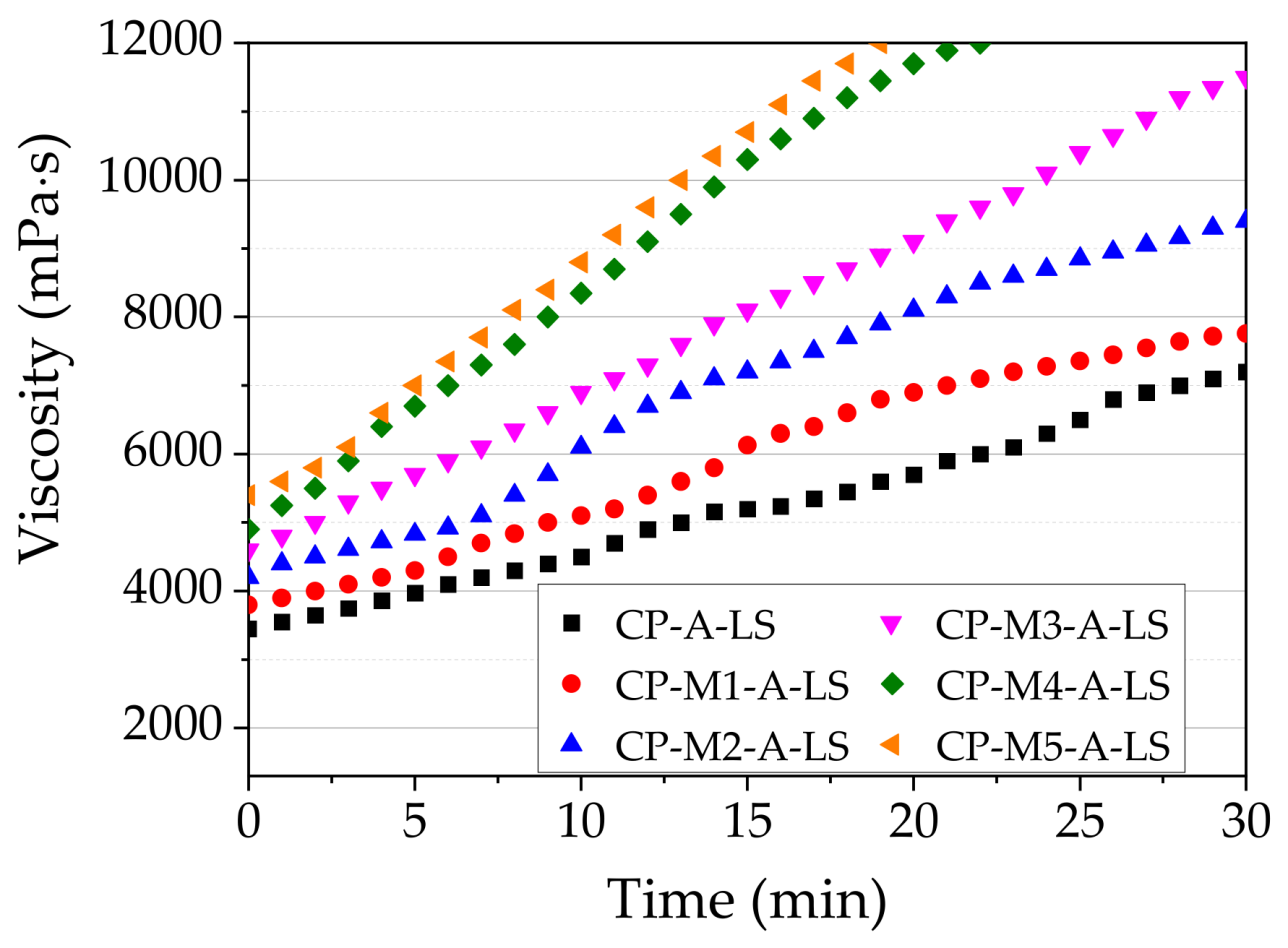
The influence of various MWCNT dosages on the dynamic viscosity of foamed cement paste in the presence of SP-LS.

When examining pastes with SP-LS (Figure 14), it is noted that the viscosity of these pastes slightly decreases with the inclusion of SP-LS. As the amount of MWCNTs (0–1.5%) in the paste increases, SP-LS reduces the initial dynamic viscosity by 12.1–7.0% compared to foamed paste CP-A, as illustrated in Figure 13. Research [20] indicates that SP reduces the viscosity of pastes containing AEA, causing compaction. AEA dosage increases with SP incorporation, resulting in coarser air bubbles than those produced by AEA alone. SP also reduces paste concrete viscosity, facilitating the escape of air bubbles. The interaction between SP and AEA adversely impacts the air-void system in fresh paste [26], associated with the negative charges from SP attachment on cement particles, which prevent AEA adsorption. Towards the end of the measurement period, the viscosity of the reference paste and pastes containing 0.094–0.375% MWCNTs is 4.5%, 1.91%, 4.1%, and 1.7% lower than that of the CP-A paste. For pastes with the highest MWCNT amounts (0.75% and 1.5%), the highest viscosity of 12,000 mPa·s was reached in 22 and 19 min, compared to 26 and 18 min in pastes without SP-LS. This indicates that for the paste with 0.375% MWCNTs, the higher viscosity was reached later by 18.5%, whereas for the paste with 1.5% MWCNTs, there was no time advantage. This could be associated with the nature of SP-LS. Research [77] suggests that lignosulphonates in cement paste accelerate ettringite formation, resulting in a rapid increase in viscosity [78].

Within 7 min of hydration, C_3_A produces big, needle-shaped ettringite that causes stiffness and pseudo-setting when it reacts instantly with gypsum (in cement) with the addition of lignosulphonates. The interaction between MWCNTs and SP-LS has been previously described [18,79]. It was found that SP-LS significantly increases the viscosity of cement paste with MWCNTs. Current viscosity test results suggest that MWCNT fibers may interact with early formed ettringite needle structures and intertwine. In contrast, the AEA admixture’s air bubble structure limits paste mobility, all contributing to the high viscosity of pastes with SP-LS.

**Figure 15:**
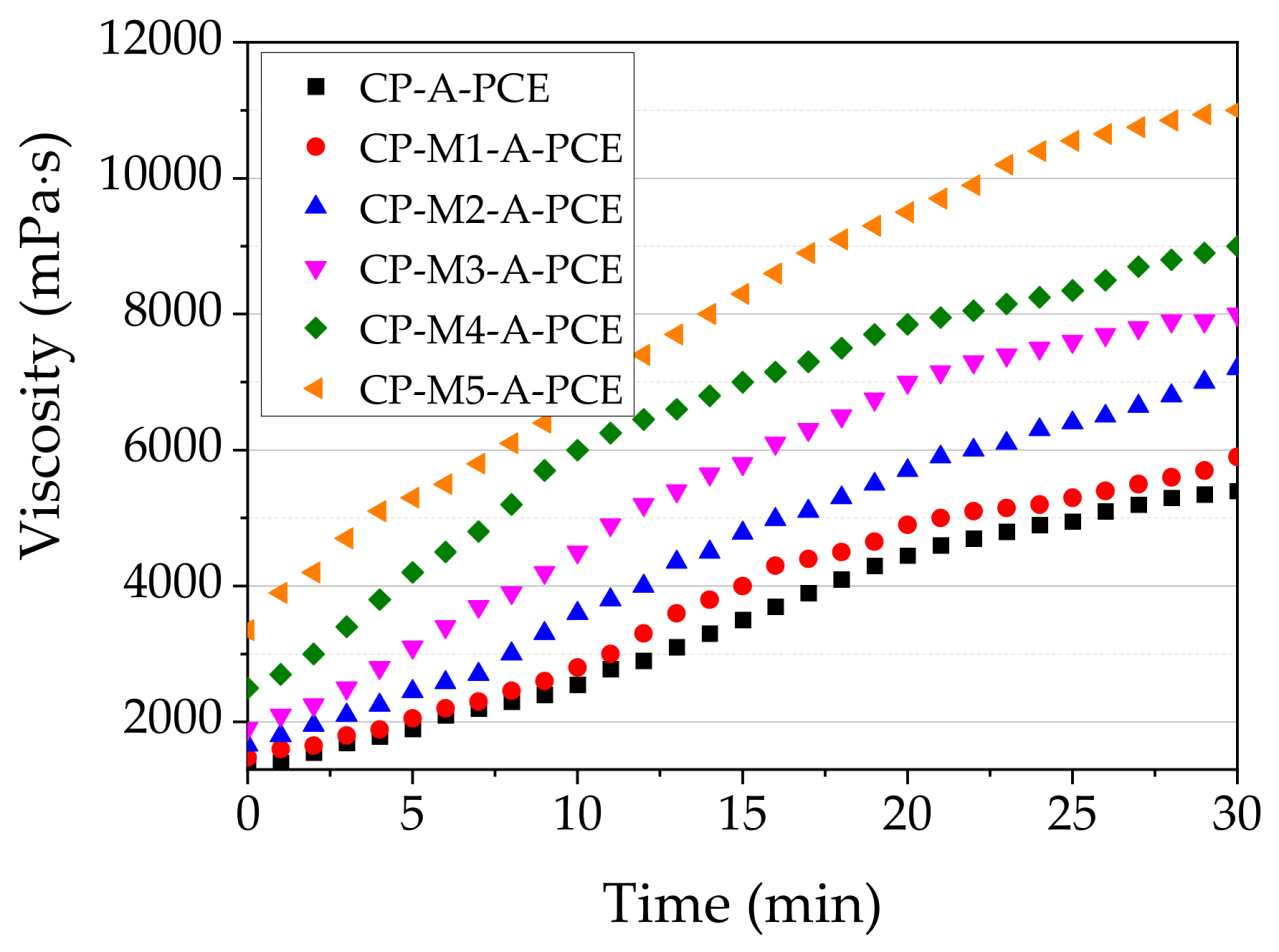
Foamed cement paste dynamic viscosity with different dosages of MWCNTs and SP-PCE.

When SP-PCE was used in the foamed pastes, as shown in Figure 15, a significant decrease in viscosity was observed compared to foamed pastes with SP-LS. According to the findings, SP-PCE most successfully lowers the initial viscosity in the reference and foamed pastes when fewer MWCNTs are present (0.094–0.375%). The initial viscosity values decreased from 20% to 48% compared to the foamed paste CP-A, as depicted in Figure 13. This reduction can be attributed to the smaller MWCNT dosage, which formed fewer air bubbles in interaction with AEA, and to the presence of SP-PCE. SP-PCE has the lowest EC values among the studied SPs and can significantly reduce cement paste EC values [79]. Its operating principle is electrostatic dispersion, which notably lowers paste viscosity. In the SP-PCE structure, polypropylene oxide side chains reduce air entrainment, enhancing the plasticizing effect on pastes [16,80,81]. SP-PCE polymers with long side chains and high carboxyl group/ester ratios can more effectively neutralize attraction forces between particles at low W/C ratios. When the MWCNT amount is relatively small, the carboxymethylcellulose content in the paste is similarly small. Air bubbles captured by MWCNT fibers and the active carboxylate groups of carboxymethylcellulose on the MWCNT surface can interact with SP-PCE carboxyl groups, forming hydrogen bonds [82].

This creates compact clusters that block water molecules from entering the cement particle, thereby increasing SP-PCE’s efficacy in the presence of AEA and MWCNTs. However, when the MWCNT amount is high, more air bubbles are trapped in the MWCNT network, making viscosity reduction more challenging due to the physical resistance from the larger number of air bubbles. At the end of the viscosity test, the viscosity of the reference and pastes with MWCNTs (up to 0.375%) is 31.3–32.7%, and for MWCNT amounts 0.75% and 1.5%, the viscosity is by 25% and 9% lower than in the same pastes CP-A without SP admixture.

**Figure 16:**
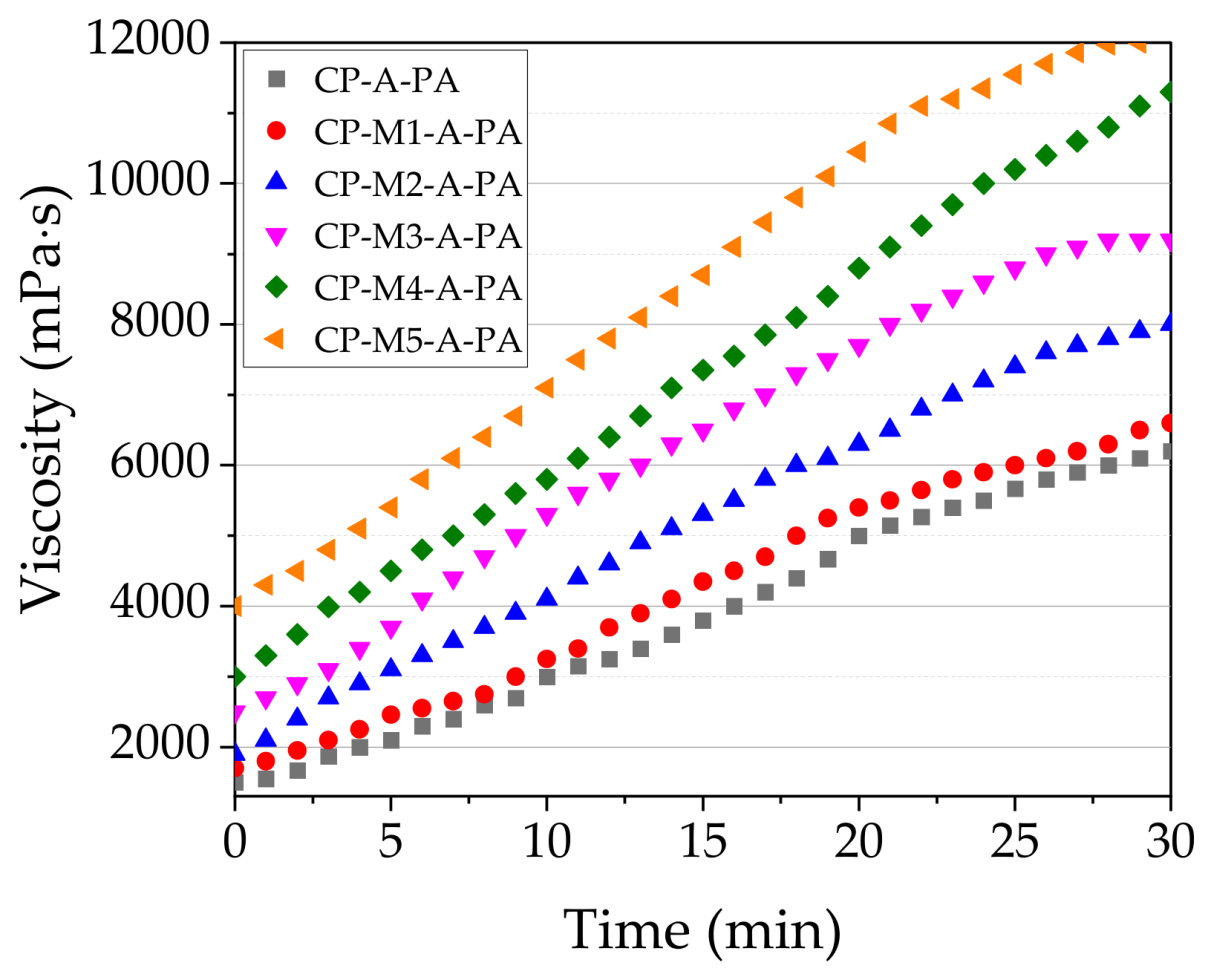
The dynamic viscosity of foamed cement paste, including SP-PA and varying dosage of MWCNTs.

Studies of foamed cement pastes with SP-PA admixture and increasing dosage of MWCNT, as shown in Figure 16, reveal that SP-PA is less effective than SP-PCE. The initial viscosity of pastes decreases from 18% to 50% compared to the foamed paste CP-A, as depicted in Figure 13. As the MWCNT dosage increases, the viscosity lowering of SP-PA, similar to SP-PCE, increases. However, the initial viscosity values of foamed pastes with SP-PA and MWCNTs (0–0.375) were 10.0–24.0%, and in the case of MWCNTs (0.75–1.5%) were 16.7% and 16.3% higher than the same foamed pastes with SP-PCE. Despite researchers [83] claiming that SP-PA has a higher charge density and better cement particle adsorption ability than SP-PCE, which was identified in zeta potential data, a higher zeta potential value for suspensions with SP-PA than with SP-PCE is observed (Figure 2 and Figure 3). The presence of AEA and MWCNTs additionally modifies the ability of SP-PA to disperse. SP-PCE has the greatest plasticizing effect across all MWCNT dosages and visibly reduces paste viscosity, so SP-PA remains viable for lower MWCNT dosages (0.094–0.188%). When SP-PA molecules adsorb onto cement particles, methacrylate molecules form linear structures (~8–9 nm), reducing flocculation between cement particles [84,85]. This demonstrates that SP-PA can effectively reduce paste viscosity with lower MWCNTs and AEA dosage. At higher MWCNT dosage, Ca^2+^ ions released from cement minerals bind to more groups on carboxymethylcellulose and SPPA methacrylate molecules, potentially increasing paste viscosity through intermolecular interactions [45]. At the end of the viscosity tests, the viscosity of the reference and pastes with 0.094–0.375% MWCNTs is 15.9–19.3%, whereas with 0.75–1.5% is up to 5.8% lower than in the same pastes without SP admixture. The highest viscosity value of 12,000 mPa·s was reached in the CP-M5-APA paste in 29 min, and in the CP-M4-A-PA paste, a high viscosity of 11,300 mPa·s was reached in 30 min.

In summary, it can be concluded that among the SP used in pastes with AEA and MWCNTs, SP-LS is the most efficient in increasing viscosity and maintaining the foamed structure of the paste. Both SPPA and SP-PCE significantly decrease paste viscosity. Specifically, SP-PCE and SP-PA reduce the initial viscosity of the pastes by 2.96–1.97 and 2.66–1.65 times, respectively, as the MWCNT dosage increases. In contrast, SP-LS only reduces viscosity by 12.1–7%. Therefore, using SP-LS in these compositions might be influenced by other factors, such as the setting time and exothermic profile. The setting time and EXO temperature tests were conducted to understand further how foamed paste with different SP interacts with MWCNTs and how they influence the hydration process.

#### 3.2.3. Fresh Cement Paste Setting Time

**Figure 17:**
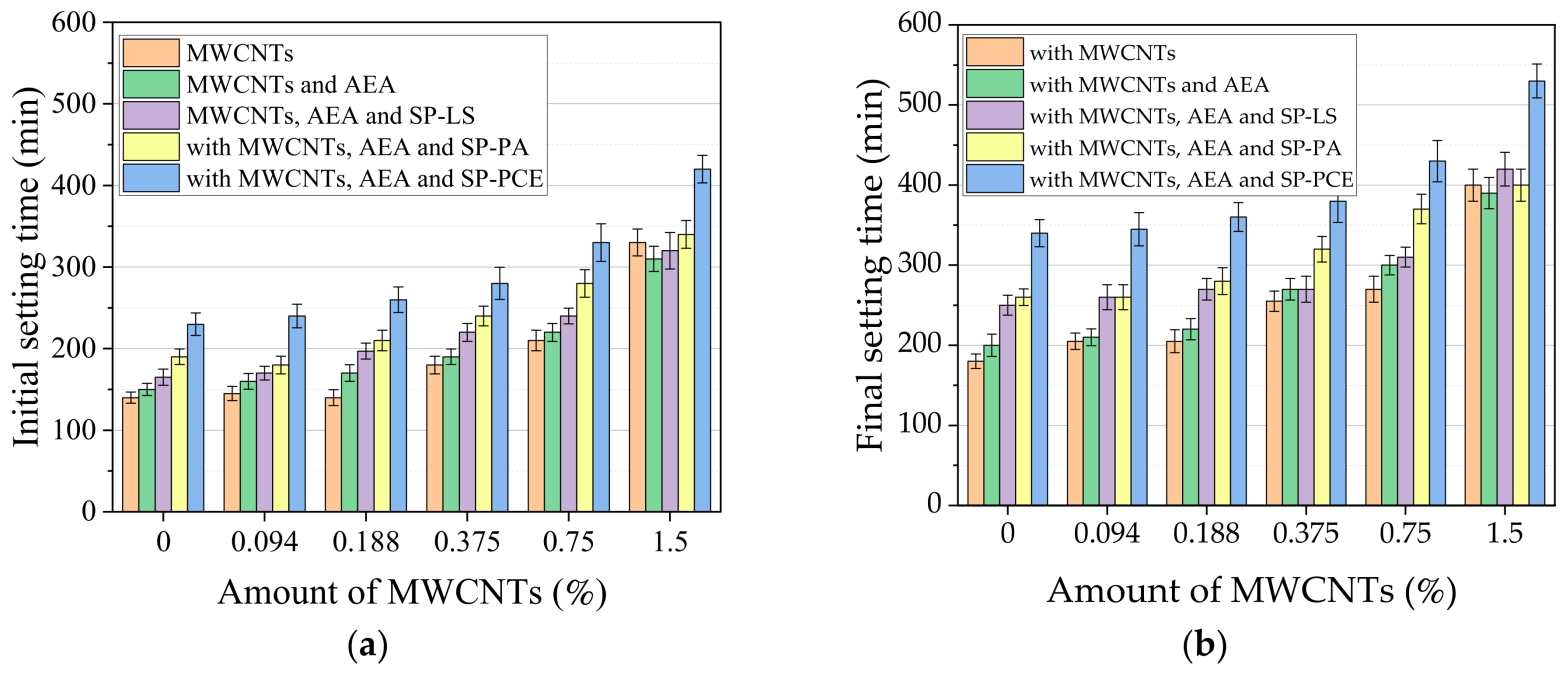
Setting time of cement pastes containing varying dosage of MWCNTs, AEA, and SP: (**a**) initial setting time, (**b**) final setting time.

As the MWCNTs dosage in the foamed paste increases to 0.094%, the initial setting time extends to 17.6%; for higher MWCNTs dosage (Figure 17a), in comparison to the MWCNT-free foamed paste, the increase is just 4.5%, and the initial setting time drops by 7.1% for the maximum MWCNTs level.

The setting time is prolonged because the (Ca^2+^) enters the solution much more slowly due to reduced cement dissolution and smaller EC values. Previous research [37] reported a similar retardation of initial and final setting times with increasing MWCNTs dosage. Research also noted that MWCNTs dosage above 0.005% significantly retard cement mineral hydration [41]. Although some studies [76,86] suggest varying influences of higher MWCNTs dosage on initial setting time, this may depend on the admixture used for MWCNTs production. SP affects the initial setting time of pastes differently.

When MWCNTs are added to the paste in levels up to 0.188%, SP-LS takes 1.35 times longer to set initially than the control foamed paste with MWCNTs, while SP-PA and SP-PCE take 1.6 and 1.73 times longer, respectively. SP-LS increases the initial setting time by 1.6 times, while SP-PA and SP-PCE increase it by 2.2 and 2.8 times, respectively, with the highest MWCNTs dosage (1.5%). The setting time can range from 1.35 to 1.73 times with MWCNTs dosage not exceeding 0.188%, and from 1.6 to 2.8 times with the highest MWCNTs dosage. This variation is related to the SP’s pH and electrical conductivity values in the solution. Table 4 shows considerable variation in ion amounts across different SP solutions, with similar EC values reported in [39]. Both alone and in combination with AEA and MWCNTs, SP-LS slightly delays the initial and final setting times due to its alkaline pH and high EC [71].

The increased initial setting time observed with SP-PCE and SP-PA can be attributed to their acidic pH, which delays the transfer of (Ca^2+^) in the solution, and possibly to the adsorption of their molecules onto anhydrous cement compounds, which delays hydration [87]. In contrast, AEA and SP-LS have an alkaline pH.

Compared to the control paste, the initial setting time is prolonged by each SP admixture when used alone, particularly in the foamed paste used alongside MWCNTs. Acidic pH admixtures in the cement paste show later setting times. With increased MWCNT dosage in foamed pastes, the final setting time increases from 201 to 388 min (Figure 17b).

A slight increase in viscosity could improve foam retention by lowering drainage and bubble rise. On the other hand, elevated viscosity might hinder workability, encourage local agglomeration, hinder mixing, and inhibit uniform bubble formation. Therefore, the best foaming performance is not always correlated with the highest viscosity.

The dosage of MWCNTs added to foamed pastes containing SPs increases the final setting time from 206 to 527 min. A noticeable extension is observed at MWCNTs dosage above 0.375%. It implies that higher MWCNTs dosage can significantly prolong the hydration process. The final setting time is increased by up to 2.7 times for SP-PCE, 2.2 times for SP-PA, and 2.0 times for SP-LS compared to SPs’ free foamed pastes. The AEA and SP’s pH and EC values impact the pastes’ viscosity, spread, and setting. The final setting time is considerably extended by SP-PCE, which is characterized by its acidic pH and the lowest EC among the studied SP admixtures. A cement paste with low-acidity or alkaline-pH admixtures sets more quickly. The setting times of the tested pastes vary widely. However, SP-LS has the most pronounced effect on setting time, which is why it was chosen for further studies of the semi-adiabatic temperature profile, reflecting the EXO reaction.

#### 3.2.4. Semi-Adiabatic Temperature-Profile Characteristics

**Figure 18:**
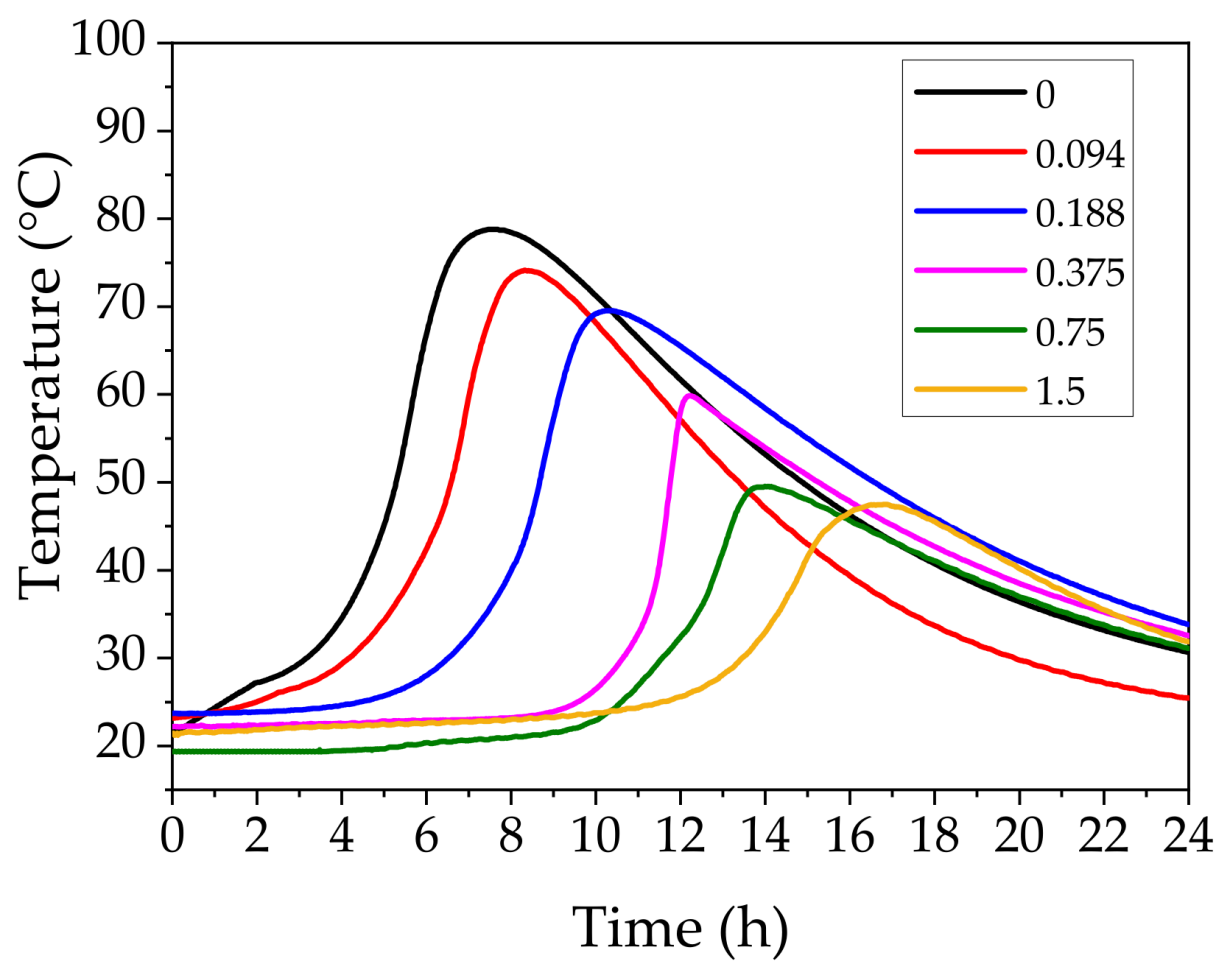
EXO temperatures change over time for foamed cement paste and foamed cement pastes containing 0.094–1.5% of MWCNTs.

**Figure 19:**
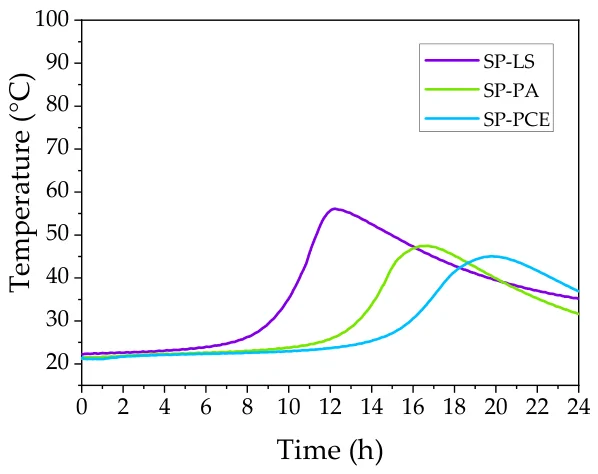
EXO temperatures change over time in foamed cement paste containing 0.375% MWCNTs and different SP (SP-LS, SP-PA, SP-PCE).

Figure 18 and Figure 19 illustrate the results of tests on fresh foamed cement pastes with various dosages of MWCNTs and fresh foamed cement pastes with selected dosages of MWCNTs and different SPs, focusing on the heat emitted during cement hydration and its influence on the temperature and timing of the maximum temperature peak. As noted in the literature [18,88], AEA can reduce the heat of hydration but has minimal impact on the timing of the EXO maximum. With the addition of MWCNTs to the foamed pastes (Figure 18), the EXO maximum temperature decreases as the MWCNT content increases, falling from 78 °C for the reference CP-A paste to 47 °C for the CP-M5-A paste containing the highest MWCNT dosage. The induction periods for the reference and the paste with the lowest MWCNTs dosage (0.094%) are 2.5 and 3.3 h, respectively, with the maximum exothermic reaction occurring at 7.1 and 8.2 h, respectively. For MWCNTs percentages of 0.188–0.375%, the induction periods are 5.2 and 9 h, respectively, and the maximum exothermic reaction occurs at 10.6 and 12.1 h, with the EXO maximum temperature dropping to 69 °C and 60 °C, respectively. The highest MWCNTs percentages (0.75–1.5%) extend the induction period to 13.6 and 17 h, respectively, and delay the EXO maximum, with temperatures dropping to 48.2 °C and 47 °C, respectively. Lower MWCNTs amounts (0.094–0.375%) decrease the EXO maximum temperature to 75 °C, 69 °C, and 60 °C, respectively, in the 3.8–23.1% range. However, the most significant temperature changes occur with higher MWCNTs amounts (0.75–1.5%), where the AEA decreases the EXO maximum temperature to 49 °C and 45 °C, respectively, by 37.2% and 42.3% compared to the CP-A paste EXO temperature. Research [37] has shown that MWCNTs reduce C_3_A hydration and decrease heat production during hydration. The clustering of MWCNTs around the cement grains may prevent C_3_A hydration. A slower or shifted early-age reaction route is consistent with the lower and extended temperature peak. However, the finding cannot be entirely attributable to decreased C_3_A hydration because the test measures specimen temperature rather than total heat release.

As shown, the most pronounced reduction in hydration temperature occurs at MWCNTs concentrations of 0.75% and 1.5%. Based on this observation, a 0.375% MWCNTs amount was selected to test the effects of SP on cement hydration.

Adding SPs further alters the EXO reaction profile of foamed cement paste with MWCNTs (Figure 19). Compared to the foamed cement paste with only 0.375% MWCNTs, the use of SP-LS shows the least impact on the induction period, the timing, and the EXO reaction temperature. The EXO maximum temperature of foamed paste with SP-LS decreases by only 4.1 °C (6.6%), and the time of the maximum exothermic reaction increases by 3.9%. However, when SP-PA and SP-PCE are used in the foamed paste, there are significant changes in the induction period duration and the EXO reaction time and temperature. The EXO maximum temperature decreases to 47 °C and 44 °C (a reduction of 21.4% and 26.3%, respectively) when SP-PA and SP-PCE are incorporated. Additionally, the maximum exothermic reaction time is delayed by 25.6% and 36.2%, respectively, when SP-PA and SP-PCE are included in the mixture.

## 4. Conclusions

The interactions between MWCNTs, AEA, and various types of SP—SP-LS, SP-PCE, and SP-PA—in suspension and cement paste were characterized using several methods, including zeta potential, pH, conductometry, viscosity, setting time, and semi-adiabatic profiles.

1. Research on MWCNTs suspension, intended for the modification of foamed cement pastes, proves that an increase in MWCNTs concentration in suspension increases the stability of the suspension, and a zeta potential was obtained. The presence of MWCNTs in foamed suspensions with SPs increases the absolute zeta potential magnitude, although the extent of the increase depended on the SP type and pH. SPs with acid pH show lower zeta potential magnitudes than those with an alkaline pH. The interaction between MWCNTs and alkali-pH AEA stabilizes the suspension, shifts the zeta potential toward negative values, and provides stronger electrostatic stabilization. A marked increase in zeta potential was observed in foamed MWCNTs with alkali-pH SP-LS; zeta potential values shift from −22 to −60, reflecting SP-LS-induced increased dispersion in this system. It can be concluded that, associated with the alkali pH of AEA and SP-LS dispersion, the stability of the MWCNTs-containing suspension increases.

2. The role of the different kinds of SP on the foamed suspension in the presence of cement and MWCNTs stability was explained. A higher MWCNTs dosage increases the initial foam volume and stabilizes it because MWCNTs induce solid particle size effects, capture air bubbles in the filament structure, and improve foam stability. The stability of foamed suspensions depends significantly on the SPs used. More significantly, among the tested SPs, the presence of alkali-pH-possessing SP-LS increased the initial volume of foamed suspension. In contrast, the presence of SP-PCE and SP-PA decreased the initial volume of foam, compared to pure foamed MWCNTs suspensions. This variation was associated with the alkaline pH and higher EC of the SP-LS solution, although differences in polymer chemistry and active-solid content may also have contributed. The research indicates that compatibility between AEA, SP-PCE, and MWCNTs is lower than with SP-LS, because the initial foam volume with SP-LS increased up to 2 times compared to the foam volumes of compositions with SP-PCE or SP-PA. This tendency is fully reflected in the results for cement paste volume.

3. pH and zeta potential results in suspension show correlation: SP-LS and AEA, possessing an alkaline pH, show a negative zeta potential region, while SP-PCE and SP-PA with acidic pH show small values in the positive zeta potential region. When AEA and SP are used together in suspensions, pH values increase, and during interaction, zeta potential values are found in the negative region. MWCNTs in the foamed suspensions decreased pH values, but in the presence of SP-LS, the pH values were the highest, as reflected in the zeta potential values, compared to suspensions with SP-PCE and SP-PA.

4. The use of MWCNTs in foamed pastes increases the pastes’ viscosity, which is more pronounced with higher MWCNTs dosage. Smaller dosages of MWCNTs trapped fewer air bubbles when interacting with AEA, resulting in a slower increase in viscosity. In pastes with higher MWCNT dosage, the viscosity increased substantially as more air bubbles were trapped between MWCNT fibers, and the MWCNTs’ reinforcing effect on air bubble walls was also observed, thereby inhibiting paste mobility. As the MWCNTs dosage in the foamed paste increases to 0.094–0.75%, the initial setting time extends to 17.6–4.5%, but for the highest MWCNTs dosage, 1.5%, the initial setting time decreases by 7.1%, compared to the MWCNTs-free foamed paste.

5. SP has varying effects on pastes’ initial setting times. The influence of SP on paste viscosity showed that SP-PCE and SP-PA are most effective at reducing viscosity. In contrast, SP-LS is most effective at supporting high viscosity and foam stability. This variation is related to the alkali pH of SP-LS. The AEA and SP’s pH and EC values impact the pastes’ viscosity, spread, and setting time. The final setting time is considerably extended by SP-PCE, which is characterized by its acidic pH and the lowest EC among the studied SP admixtures. Cement paste with low acidity or alkaline pH admixtures sets more quickly.

6. The semi-adiabatic profiles studies showed that increasing the percentage of MWCNTs in the foamed paste extended the maximum exothermic reaction time while lowering the peak temperature. Lower MWCNT dosage (0.094–0.375%) decrease the EXO maximum temperature from 75 °C to 60 °C; higher MWCNTs dosage (0.75–1.5%) decrease it more significantly, up to 45 °C, and achieve up to a 42.3% decrease in the EXO maximum temperature. The addition of SP-LS to foamed paste with a stable MWCNT dosage (0.375%) shows the least impact on the induction period and the timing and temperature of the EXO reaction. The EXO maximum temperature of foamed paste with SP-LS decreases by only 4.1 °C (6.6%), and the time of the maximum exothermic reaction increases by 3.9%. However, when SP-PA and SP-PCE are used in the foamed paste, the reduction in the EXO maximum temperature reaches 21.4% and 26.3%, respectively, and the time to the maximum exothermic reaction is delayed by 25.6% and 36.2%, respectively.

7. The current findings show that AEA and MWCNTs-containing pellets interact, as evidenced by variations in density, viscosity, foam retention, and the absolute magnitude of the zeta potential. Multiple mechanisms could be involved simultaneously. The presence of MWCNTs, especially when the concentration in foamed suspensions is increased, increases zeta potential, improves the dispersion of MWCNTs, and increases the stability of the foamed suspension. The high-aspect-ratio MWCNT-containing filaments may physically prevent bubble migration and coalescence, but the anionic AEA may adsorb on solid surfaces or interact with Ca^2+^ containing species. Furthermore, the commercial granules’ CMC may alter surfactant adsorption and raise water-phase viscosity.

8. Nevertheless, the molecular interaction mechanism cannot be clearly determined from the available data because adsorption measurements and interfacial-tension investigations were not carried out.

9. The present study demonstrates the potential to improve the stability of foamed cement pastes, which is essential for the broader application of lightweight composites. The findings identify promising fresh-state compositions for further investigation. However, their suitability for practical applications in lightweight composites must be confirmed by evaluating hardened density, mechanical strength, thermal conductivity, pore structure, and durability.

## Figures and Tables

**Table 1:** SP and AEA electrical conductivity and pH parameters.

Additive Type				
	SP-LS	SP-PA	SP-PCE	AEA
pH	8.46	6.01	4.60	8.05
EC, S/m	2011 × 10^−4^	551 × 10^−4^	367 × 10^−4^	367 × 10^−4^

**Table 2:** pH and electrical conductivity characteristics of MWCNTs in water solution.

Dosage of MWCNTs in Water Solution, %						
	0	0.094	0.188	0.375	0.75	1.5
pH	6.89	6.75	6.60	6.42	6.07	5.78
EC, S/m	15 × 10^−4^	166 × 10^−4^	196 × 10^−4^	250 × 10^−4^	318 × 10^−4^	490 × 10^−4^

## Data Availability

The original contributions presented in this study are included in the article. Further inquiries can be directed to the corresponding author.
